# Action Potential Initiation in Neocortical Inhibitory Interneurons

**DOI:** 10.1371/journal.pbio.1001944

**Published:** 2014-09-09

**Authors:** Tun Li, Cuiping Tian, Paolo Scalmani, Carolina Frassoni, Massimo Mantegazza, Yonghong Wang, Mingpo Yang, Si Wu, Yousheng Shu

**Affiliations:** 1 Institute of Neuroscience and State Key Laboratory of Neuroscience, Shanghai Institutes for Biological Sciences, Chinese Academy of Sciences, and University of Chinese Academy of Sciences, Shanghai, China; 2 U.O. of Neurophysiopathology and Diagnostic Epileptology, Foundation Istituto di Ricerca e Cura a Carattere Scientifico (IRCCS) Neurological Institute Carlo Besta, Milano, Italy; 3 U.O. of Clinical Epileptology and Experimental Neurophysiology, Foundation Istituto di Ricerca e Cura a Carattere Scientifico (IRCCS) Neurological Institute Carlo Besta, Milano, Italy; 4 Institute of Molecular and Cellular Pharmacology (IPMC), Laboratory of Excellence Ion Channel Science and Therapeutics (LabEx ICST), CNRS UMR7275 and University of Nice-Sophia Antipolis, Valbonne, France; 5 State Key Laboratory of Cognitive Neuroscience and Learning and IDG/McGovern Institute for Brain Research, School of Brain and Cognitive Sciences, Beijing Normal University, Beijing, China; 6 Center for Collaboration and Innovation in Brain and Learning Sciences, Beijing Normal University, Beijing, China; ICM - Institut du Cerveau et de la Moelle épinière Hôpital Pitié-Salpêtrière, France

## Abstract

Action potential (AP) generation in inhibitory interneurons is critical for cortical excitation-inhibition balance and information processing. However, it remains unclear what determines AP initiation in different interneurons. We focused on two predominant interneuron types in neocortex: parvalbumin (PV)- and somatostatin (SST)-expressing neurons. Patch-clamp recording from mouse prefrontal cortical slices showed that axonal but not somatic Na^+^ channels exhibit different voltage-dependent properties. The minimal activation voltage of axonal channels in SST was substantially higher (∼7 mV) than in PV cells, consistent with differences in AP thresholds. A more mixed distribution of high- and low-threshold channel subtypes at the axon initial segment (AIS) of SST cells may lead to these differences. Surprisingly, Na_V_1.2 was found accumulated at AIS of SST but not PV cells; reducing Na_V_1.2-mediated currents in interneurons promoted recurrent network activity. Together, our results reveal the molecular identity of axonal Na^+^ channels in interneurons and their contribution to AP generation and regulation of network activity.

## Abstract 2

Sodium channels add variety to inhibitory interneurons Different populations of inhibitory interneurons in the cerebral cortex express distinct subtypes of sodium channels, resulting in diverse action potential thresholds and network excitability.

## Author Summary

Inhibitory interneurons in the cerebral cortex are diverse in many respects. Here, we examine whether this diversity extends to the composition of ion channels along the axon, which might determine the neurons' excitability. We performed patch-clamp recordings from cortical interneuron axons in brain slices obtained from two transgenic mouse lines. In each mouse line, distinct populations of inhibitory interneurons—those that express parvalbumin (PV) or those that express somatostatin (SST)—were labeled with green fluorescent protein to allow visualization. We show that action potentials initiate at the axon initial segment (a specialized region of the axon closest to the cell body) in both cell types, but SST neurons have a higher action potential threshold than PV neurons because their sodium channels require a greater degree of depolarization to be fully activated. At the molecular level, we found that the population of sodium channels in SST neurons requires a larger depolarization because it has a more mixed composition of high- and low-threshold sodium channel subtypes. In summary, this study reveals diversity in the molecular identity and voltage dependence of sodium channels that are responsible for initiating action potentials in different populations of interneurons. In addition, the presence of a particular subtype of sodium channel—Na_V_1.2—in inhibitory interneurons might explain why loss-of-function mutations in this channel result in epilepsy.

## Introduction

In general, synaptic inputs that arrive at the dendrites and the cell body of a neuron interact with intrinsic membrane properties and cause the generation of the main output signal, the action potential (AP), at the axon initial segment (AIS) [1]–[5]. Previous modeling, immunostaining, and electrophysiological studies suggest that a high density of Na^+^ channels at the AIS determines the lowest threshold for AP initiation [6]–[9]. A recent study in cortical pyramidal cell (PC) further demonstrated that the accumulation of Na_V_1.6, a low-threshold Na^+^ channel subtype, at the distal end of AIS determines AP initiation, whereas the accumulation of high-threshold Na_V_1.2 at the proximal AIS regulates AP backpropagation to the soma and dendrites [10]. In addition, recent studies also showed that the location of Na_V_1.6 and the whole AIS are subjected to regulation by neuronal activity [11],[12]. These features, together with selective distribution of certain types of K^+^ and Ca^2+^ channels at the AIS, may contribute to the generation and regulation of neuronal signaling [13]–[17]. The cerebral cortex contains not only excitatory PCs but also their counterparts, the inhibitory interneurons. The capability of initiating APs, particularly with precise timing, in these interneurons is critical for maintaining the excitation-inhibition balance and shaping the output signal of their target neurons. However, the underlying mechanisms for AP initiation in inhibitory interneurons remain poorly understood.

Previous studies revealed the expression of Na_V_1.1 channels at the AIS of inhibitory interneurons but not in excitatory PCs [18],[19]. Mutations of Na^+^ channels have been identified in several types of epilepsy [20]. Loss-of-function mutations in *Scn1a* gene encoding the Na_V_1.1 α subunit can result in a reduction of excitability in inhibitory neurons but an increase in network activity, leading to severe epilepsy in human patients and animal models [21]–[24]. Interestingly, both gain- and loss-of-function mutations of the *Scn2a* gene encoding the Na_V_1.2 α subunit can be associated with some forms of epilepsy [25]–[29]. Intellectual decline and idiopathic autism were also found in patients with *Scn2a* mutations [28],[30]. Because PCs express Na_V_1.2 channels, gain-of-function mutations may cause hyperexcitability of these excitatory neurons and thus increase epilepsy susceptibility in patients. However, it remains unclear why loss-of-function mutations also link to the generation of epilepsy. Recent studies on mutations of the *Scn8a* encoding Na_V_1.6 revealed a similar variability in functional effects with consequent difficulties in identifying clear pathomechanisms [31],[32].

Cortical inhibitory interneurons show great diversity in their morphology, firing patterns, synaptic plasticity, and gene expression [33],[34]. Among them, the parvalbumin (PV)-containing fast-spiking and somatostatin (SST)-containing low-threshold spiking neurons are the most abundant interneuron subtypes [35]–[37]. Apart from the difference in firing patterns, they respond to stimuli with different latency and duration. Although PV neurons show a delay-type firing pattern with near-threshold current injections [38],[39], they discharge APs with precise timing at the beginning of an extracellular stimulus train with high intensity [40]. In contrast, SST neurons wait until the late phase of the stimulus train to enter a persistent firing mode [40]. Previous studies attribute these differences to passive cable properties and short-term plasticity in excitatory synapses onto PV (synaptic depression) and SST neurons (synaptic facilitation) [41]–[43]. Distinct channel distribution patterns in dendrites of these neurons may also contribute. In comparison with PV neurons, SST neurons express a relatively high density of Na^+^ channels in their dendrites, which can boost synaptic responses in distal dendrites and contribute to the distinct paired-pulse facilitation in excitatory synapses onto SST neurons [44],[45]. Considering that synaptic events occurring in the dendrites will eventually sum up in the axon to generate APs, we sought to investigate whether the diversity of inhibitory interneurons also extends to the axonal level.

We performed recording from axonal blebs, the resealed cut ends formed during slicing procedures [10],[46] from PV and SST neurons, to investigate the biophysical properties of Na^+^ channels in the AIS or adjacent axonal regions, and carried out immunostaining to reveal their molecular identity. Our results show that AIS Na^+^ channels in SST neurons activate at higher (more depolarizing) membrane potential (*V*_m_) levels than those in PV neurons, corresponding well with AP threshold differences in the two types of neurons. As in PCs, segregation of Na^+^ channel subtypes also occurs at the AIS of both PV and SST neurons. A more mixed distribution of high- and low-threshold channel subtypes in the SST axons may increase the threshold for AP initiation. Surprisingly, Na_V_1.2 channels were found accumulated at the proximal AIS of SST but completely absent in PV neurons. Further experiments suggested that interneuronal Na_V_1.2 channels play an important role in shaping network activity.

## Results

### Difference in AP Threshold

**Figure 1:**
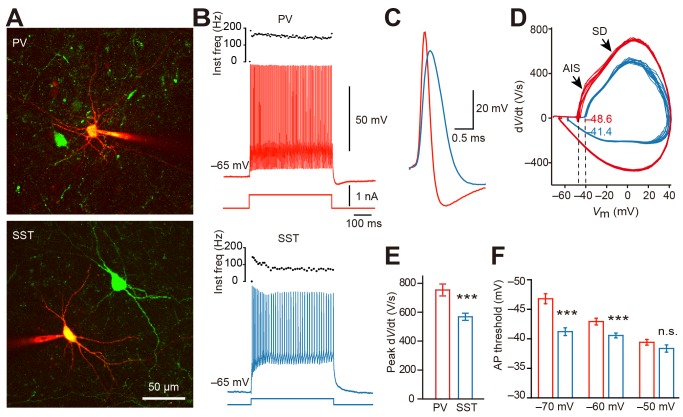
Difference in voltage thresholds of APs in PV and SST neurons. (A) Projection of two-photon images showing recordings from PV-positive (top, B13 mouse) and SST-positive (bottom, GIN mouse) neurons in prefrontal cortical slices. Cells were loaded with Alexa Fluor 594 (red) through patch pipettes. (B) Firing patterns of the two recorded cells shown in (A). Traces are color-coded (PV, red; SST, blue). (C) Overlaid APs from PV and SST neurons. Note the difference in AP waveforms. (D) Phase plots of PV and SST APs evoked by brief current injections. Note the AIS and SD potential components. (E) Difference in peak amplitudes of d*V*/dt. (F) Dependence of AP thresholds on *V*_m_ levels. For (E) and (F), *** *p*<0.001. Error bars represent s.e.m.

We performed whole-cell recordings from inhibitory interneurons that contained PV or SST in prefrontal cortical slices (Figure 1A). We used two lines of transgenic mice, B13 and GIN mice, with GFP selectively expressed in PV- and SST-containing neurons, respectively [47],[48]. These neurons showed a significant difference in their resting *V*_m_ (PV, −71.0±0.4 mV, *n* = 13; SST, −58.2±1.0 mV, *n* = 11; *p*<0.001) and varied in intrinsic properties. The input resistances of PV and SST neurons were 90.0±8.0 and 284.1±19.2 MΩ (*p*<0.001), respectively. In response to step current injections (500 ms in duration), PV cells exhibited typical nonadapting high-frequency discharges, whereas SST neurons displayed apparent frequency adaptation (Figure 1B). APs in PV cells showed much shorter duration than those in SST cells (half-width, 0.25±0.02 versus 0.41±0.01 ms, *n* = 16 for both; *p*<0.001; Figure 1C). The threshold current (500-ms long pulses) for AP generation was 347.3±43.1 pA in PV (*n* = 14), significantly greater than that in SST neurons (39.8±6.0 pA, *n* = 12; *p*<0.001). As reported previously [38],[39], PV neurons discharged with a prominent delay with near-threshold current stimulation, resulting from the activation of K_V_1 channels. The delay of the first AP to the stimulation onset was 299±37 ms. In the presence of 100 nM α-Dendrotoxin (α-DTx), a potent K_V_1 channel blocker, the delay could be reduced to 65±15 ms (*n* = 10, *p*<0.001; Figure S1A and C). The delay-type firing pattern was not observed in SST neurons, in which a prolonged current pulse produced a depolarizing ramp before the first AP (Figure S1B). The duration of this ramp showed no significant change after the application of α-DTx (160±34 versus 140±40 ms, *n* = 10, *p* = 0.70; Figure S1C).

APs evoked from a holding *V*_m_ of −70 mV were used for the measurement of voltage threshold (see Materials and Methods). When the AP threshold was determined as the voltage at which the derivative of *V*_m_ surpassed 20 V/s, the average AP threshold in PV was −47.8±0.7 mV (*n* = 22), ∼7 mV lower than that of SST neurons (−41.1±0.5 mV, *n* = 24; Figure 1D–F). Similar results were obtained when the AP threshold was defined as the voltage at which the second derivative of *V*_m_ reached the peak (PV, −46.4±0.9 mV, *n* = 15; SST, −40.8±0.6 mV, *n* = 16; *p*<0.001).

Considering that subthreshold *V*_m_ depolarization might alter the AP threshold, we next measured the threshold (d*V*/dt = 20 V/s) at depolarizing *V*_m_ levels (Figure 1F). We injected constant currents to maintain the *V*_m_ at −60 and −50 mV and brief pulses to evoke APs. At −60 mV, the average AP threshold in PV was −42.9±0.6 mV (*n* = 15), significant lower than that in SST neurons (−40.6±0.4 mV, *n* = 13; *p*<0.05). Interestingly, no significant difference in the threshold from a holding *V*_m_ of −50 mV was observed (−39.4±0.5 in PV versus −38.4±0.6 mV in SST). These results indicate that the AP threshold is lower in PV interneurons than in SST interneurons at *V*_m_ levels lower than −50 mV (Figure 1F).

Previous studies showed that the presence of K_V_1 channel blocker α-DTx substantially hyperpolarized the threshold of the first AP with near-threshold current stimulation [38],[39]. We observed a similar effect of α-DTx in PV neurons (−34.7±1.5 in control versus −42.5±2.1 mV in α-DTx, *n* = 10, *p*<0.01, Figure S1C) but not in SST neurons (−36.9±1.3 versus −38.0±2.0 mV, *n* = 10, *p* = 0.63; Figure S1C). In this study, we compared the threshold of APs evoked by brief (2 ms in duration) and high-intensity stimulations in the two neuronal types. With this stimulation protocol, AP threshold was not affected by the application of α-DTx (for PV, −47.3±1.1 in control and −49.1±1.6 mV in α-DTx, *n* = 10, *p* = 0.38; for SST, −37.9±1.2 versus −38.2±2.1 mV, *n* = 10, *p* = 0.90; Figure S1D). These results support the notion that PV neurons respond preferentially to synaptic inputs that are large and fast enough to “outrun” K_V_1 activation [38],[39].

### AP Initiation Site

**Figure 2:**
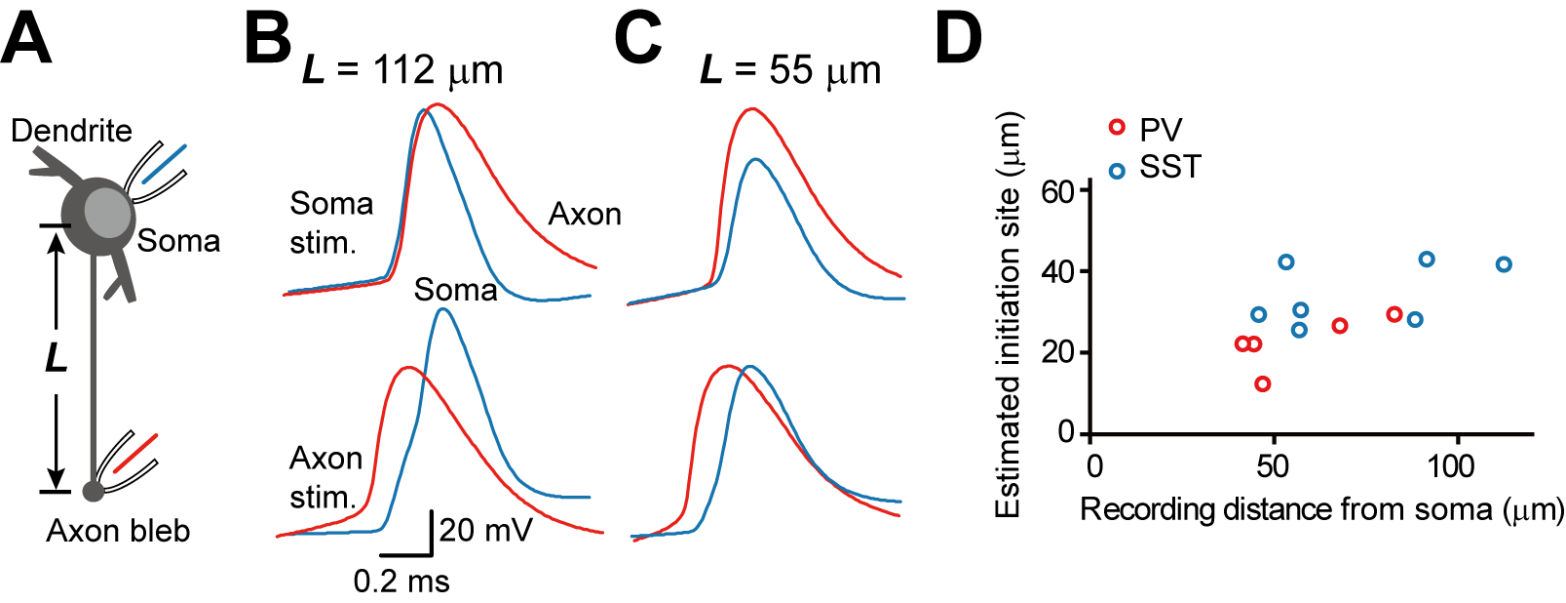
Estimation of AP initiation site at the AIS of PV and SST neurons. (A) Schematic diagram of simultaneous recording from the soma and the axon bleb. (B) Example recording from a PV neuron with an axon bleb formed 112 µm away from the soma. APs evoked by current injections at the soma (top) or axonal bleb (bottom). When the soma was stimulated, somatic AP generated earlier than axonal AP; in contrast, when the axon bleb was stimulated, axonal AP occurred earlier. (C) Similar to (B) except that the recording distance was 55 µm. Note that axonal APs preceded somatic APs in both conditions. (D) The estimated initiation sites (see Materials and Methods) in PV were more proximal than that in SST neurons.

As in PCs [10], phase plots of APs in both cell types showed two obvious components in the rising phase (Figure 1D), indicating the occurrence of AIS potential and somatodendritic (SD) potential, a phenomenon that suggests an initiation site at the AIS [49]. We next performed simultaneous recording from the soma and the bleb to estimate the AP initiation site as described before [1]. Because blebs were cut ends of the axons, they were located on the slice surface. We recorded those GFP-positive blebs connected to their soma with traceable axon trunks under the fluorescence microscope. In our experiments, we found axons emerged directly from the soma in 86.7% PV (*n* = 26/30) and 80.6% SST neurons (*n* = 25/31) examined, whereas the remaining cells had an axon emerging from their dendrites (Figure S2). We therefore only focused on cells with soma-originated axons in the following experiments unless otherwise stated. We analyzed the timing of somatic and axonal APs evoked by either somatic (initiated at the normal AIS site) or axonal stimulation (initiated at axonal blebs) (Figure 2A–C). The velocity of antidromic APs from axonal blebs was similar in the two neuron types (0.38±0.07 m/s in SST, *n* = 7; 0.46±0.06 m/s in PV, *n* = 5, *p* = 0.42). By assuming the velocities of AP propagation in orthodromic and antidromic directions were equal, we found that the estimated AP initiation site in SST neurons (34.3±2.9 µm away from the soma, *n* = 7) was significantly more distal than that in PV neurons (22.5±2.9 µm, *n* = 5, *p*<0.05; Figure 2D). Because of the great capacitance load at the soma, AP backpropagation from AIS to soma should be slower than conduction along the main axonal trunk, and the true initiation site should be closer to soma than the estimated location; however, the estimated length represents the upper limit of the distance between the soma and the initiation site.

To further confirm that the AIS has the lowest threshold for AP initiation, we next puffed TTX at the perisomatic region or the AIS and monitored changes in AP threshold. We monitored changes in AP waveform within miliseconds after puff. Within this short period of time, TTX blocked local channels in the vicinity of pipette tip. In PV neurons, application of 10 µM TTX at the perisomatic region substantially reduced the peak amplitude and peak d*V*/dt of somatic APs (567±61 in control versus 333±43 V/s with TTX; *p*<0.01), but showed no significant decrease in AP threshold (−50.4±1.6 mV versus −49.1±1.4 mV, *n* = 5, *p* = 0.54; Figure S3). In contrast, puffing TTX at the AIS caused a significant increase in AP threshold (−50.4±0.8 versus −39.9±1.7 mV, *n* = 7, *p*<0.001), whereas the peak d*V*/dt showed no significant change (557±29 versus 468±38 V/s, *n* = 7, *p* = 0.08; Figure S3). Similar results were obtained from SST neurons: TTX application at the AIS (but not at the perisomatic region) increased the AP threshold from −43.5±1.1 to −35.0±1.4 mV (*n* = 5, *p*<0.01; Figure S3). Together, the results indicate that, similar to PCs, AIS determines the lowest threshold for AP initiation in the two types of interneurons, and the initiation site in SST is more distal than that in PV cells.

### Somatic Na^+^ Channels

**Figure 3:**
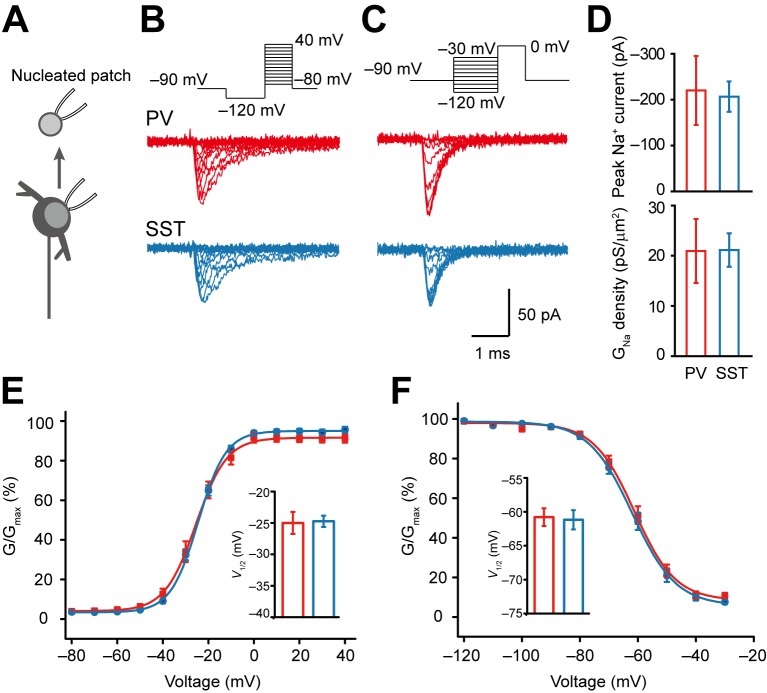
Voltage dependence of somatic Na^+^ channels. (A) Schematic diagram of recording from somatic nucleated patch (i.e., giant outside-out patch of somatic membrane). (B) Example current traces evoked by activation voltage commands (top) in PV and SST nucleated patches. (C) Current traces evoked by the test pulse (0 mV) in the voltage protocol for channel inactivation. (D) Comparison of averaged peak Na^+^ currents and conductance density in nucleated patches. Error bars represent s.e.m. (E and F) Activation and availability curves of somatic Na^+^ currents in PV (red) and SST neurons (blue). (Insets) Comparison of the activation and inactivation *V*_1/2_, showing no difference between the two cell types. Error bars represent s.e.m.

To examine the contribution of somatic Na^+^ channels to the generation of APs, we next performed voltage-clamp experiments in nucleated patches of PV and SST neurons (Figure 3). For the voltage dependence of channel activation, nucleated patches were held at −90 mV and Na^+^ currents were evoked by a series of 30-ms-long test pulses from −80 to +40 mV after a prepulse at −120 mV (50 ms in duration; Figure 3B and C).

The peak amplitude of Na^+^ currents was −220±75 and −206±33 pA in PV (*n* = 12) and SST neurons (*n* = 14), respectively (Figure 3D). Calculation of the current and the conductance density revealed that channel density in PV was similar to that in SST neurons (0.69±0.21 in PV versus 0.70±0.11 pA/µm^2^ in SST; 20.9±6.4 versus 21.1±3.3 pS/µm^2^, *p* = 0.98; Figure 3D). Somatic Na^+^ channels in the two cell types shared similar voltage-dependent properties. The minimal activation voltages (the *V*_m_ level at which the peak conductance reached 10% of its maximum value) were −43.4±1.4 in PV and −40.9±0.9 mV in SST neurons (*p* = 0.14). The half-activation voltages (V_1/2_) were −25.0±1.8 and −24.7±0.9 mV (*n* = 12 PV and 14 SST neurons; *p* = 0.89), and the slope factors of activation curves were 6.5±0.6 and 6.1±0.2, respectively (*p* = 0.51; Figure 3B and E). To examine the voltage dependence of steady-state inactivation, we applied a series of 50-ms-long prepulses from −120 to −30 mV and obtained Na^+^ currents by stepping the *V*_m_ from the level of prepulse to 0 mV. In both PV and SST neurons, the inactivation curves were well fitted by Boltzmann functions and overlapped with each other. The V_1/2_ of the inactivation curves were −60.8±1.3 (*n* = 12) and −61.2±1.4 mV (*n* = 14, *p* = 0.84), and the slope factors were 6.2±0.3 and 6.7±0.2 (*p* = 0.17) in PV and SST patches, respectively (Figure 3C and F). Interestingly, these voltage-dependent properties in the two types of interneurons were also similar to those observed in somatic patches of PCs. The V_1/2_ of activation and inactivation curves in PC somatic Na^+^ channels were −23.6±2.4 and −62.7±2.5 mV, respectively (*n* = 6), showing no significant difference from those in the two types of interneurons (*p* = 0.86 for activation and 0.76 for inactivation V_1/2_, one-way ANOVA). In agreement with previous findings [50], these results indicate similar voltage-dependent properties of somatic Na^+^ channels in PCs and interneurons.

We next compared the time course of Na^+^ currents induced at −20 mV in PV and SST somatic nucleated patches. The activation time constants were 0.15±0.02 in PV (*n* = 12) and 0.12±0.01 ms in SST neurons (*n* = 15), showing no significant difference (*p* = 0.08). The decay of Na^+^ currents was slightly slower in PV, and the decay time constant obtained from fitting the decay phase with a single exponential function was 1.14±0.13 in PV and 0.81±0.05 ms in SST neurons (*p*<0.05).

Together, these results reveal similar voltage dependence of activation and inactivation of somatic Na^+^ channels in the two types of interneurons, indicating that the difference in AP thresholds of PV and SST neurons may not result from gating properties of somatic Na^+^ channels.

### Axonal Na^+^ Channels

**Figure 4:**
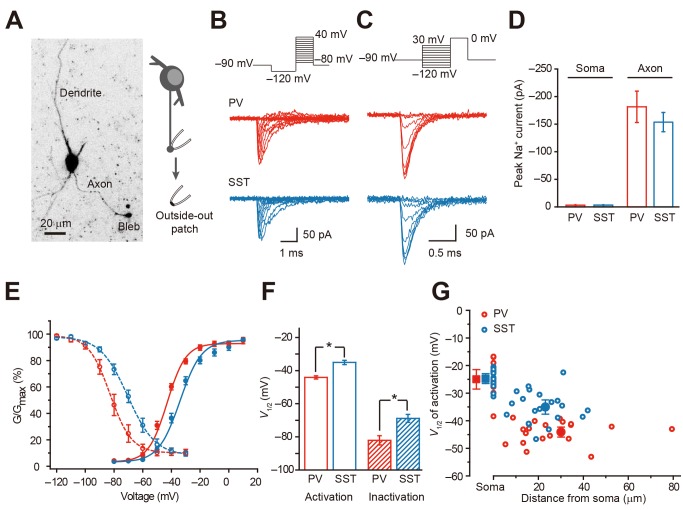
Difference in voltage dependence of axonal Na^+^ channels. (A) Projection of two-photon *z*-stack images of a GFP-positive PV neuron (left, black/white inverted). Note the axon bleb. (Right) Schematic diagram of the outside-out recording from patches excised from axon blebs. (B and C) Example current traces evoked by activation and inactivation voltage commands in PV and SST axonal patches. (D) Group data showing no significant difference in peak amplitude of axonal Na^+^ currents. However, in both types of neurons, the peak amplitudes of Na^+^ currents in outside-out patches of the axon were much greater than those in the soma. Error bars represent s.e.m. (E) Activation and availability curves for axonal Na^+^ currents. (F) Comparison of *V*_1/2_ of activation and inactivation in the two cell types. * *p*<0.05. Error bars represent s.e.m. (G) The *V*_1/2_ of activation was plotted as a function of recording distances from the soma. The average *V*_1/2_ (±s.e.m.) of somatic and axonal Na^+^ currents is shown for comparison.

We next performed similar experiments to examine the gating properties of axonal Na^+^ channels in PV and SST neurons (Figure 4). We searched for axonal blebs containing GFP on the surface of cortical slices. Whole-cell recording and then outside-out patch recording could be achieved from these blebs (Figure 4A). Using similar voltage commands used for somatic nucleated patches, we compared the current density and voltage-dependent properties of axonal Na^+^ channels in the two types of interneurons (Figure 4B and C).

The amplitude of Na^+^ currents peaked between −30 and −20 mV, and then became smaller and reversed at more depolarized potentials in both PV and SST neurons. The average peak amplitude was −181±29 pA in PV neurons (*n* = 19), similar to that observed in SST neurons (154±18 pA, *n* = 20, *p* = 0.41; Figure 4D). Because we were using patch pipettes with identical tip sizes, the similarity in peak amplitude of Na^+^ currents reflected a comparable channel density in PV and SST axons. To compare the channel density between soma and axon, we also performed recordings from regular outside-out patches excised from the soma. The average peak amplitudes of Na^+^ currents in somatic patches were −3.0±0.7 pA in PV (*n* = 19) and −3.1±0.7 pA in SST neurons (*n* = 13, *p* = 0.92; Figure 4D). These results suggest that the channel density at the axon is approximately 60- (in PV) and 50- (in SST) fold greater than that at the soma.

In agreement with the results showing the AIS had the lowest threshold for AP initiation, we found axonal channels in both PV and SST neurons activated at lower *V*_m_ levels than somatic channels (Figure 4E–G); however, to our surprise, axonal Na^+^ channels in PV neurons activated at a lower potential than SST neurons, as indicated by a left shift of the activation curve (Figure 4E). The minimal activation voltages were −62.1±1.8 and −54.6±1.1 mV for PV (*n* = 18) and SST neurons (*n* = 22, *p*<0.001), respectively. The averaged V_1/2_ of activation was −43.3±0.9 mV (*n* = 19) in PV, ∼7 mV lower (more hyperpolarizing) than that in SST neurons (−36.3±1.0 mV, *n* = 20, *p*<0.001; Figure 4F). However, the slope factors of activation curves (7.19±0.63 for PV and 6.56±0.34 for SST, *p* = 0.37) showed no significant difference. We found no significant difference in the V_1/2_ between layer 2/3 and layer 5 and pooled the results across layers. For PV neurons, the V_1/2_ for activation was −43.9±0.8 in layer 2/3 (*n* = 9) and −44.3±1.7 mV in layer 5 (*n* = 10, *p* = 0.86). For SST neurons, the V_1/2_ for activation was −36.5±1.5 in layer 2/3 (*n* = 11) and −34.4±2.4 mV in layer 5 (*n* = 8, *p* = 0.49). Again, we also examined the steady-state inactivation of axonal Na^+^ channels. The averaged V_1/2_ and slope factor were −82.1±2.8 mV and 6.37±0.96 in PV (*n* = 6) and −68.9±2.4 mV and 8.42±0.48 in SST neurons (*n* = 16, *p*<0.01 for V_1/2_ and *p* = 0.05 for slope factor; Figure 4F), respectively. We also performed outside-out patch recording on blebs of PCs (axon length >150 µm) and found that, in agreement with previous findings in rat PCs, the average V_1/2_ of activation and inactivation were −39.5±1.8 and −82.8±2.6 mV, respectively (*n* = 10). Interestingly, these values showed no significant difference from those in PV axons (*p* = 0.05 and 0.85 for activation and inactivation, respectively), suggesting that Na^+^ channel subtypes in PV axons (but not SST axons) share similar voltage-dependent properties with those in PC axons. Plotting the V_1/2_ of activation as a function of the distance from soma revealed a sharp decrease (hyperpolarization) in V_1/2_ at the AIS (0–50 µm) in both PV and SST neurons (Figure 4G). However, unlike that in PC, this decrease was less distance-dependent, possibly due to lack of recordings near the soma. Consistent with the average data, the V_1/2_ of SST axonal channels was substantially more depolarized than that of PV channels (Figure 4G). In accordance with the difference in activation V_1/2_ between somatic and axonal channels shown in Figure 4G, the difference in inactivation V_1/2_ between soma and axon was also prominent in both neuronal types: −60.8±1.3 mV (*n* = 12) for the soma and −82.1±2.8 mV (*n* = 6, *p*<0.001) for the axon in PV neurons; −61.2±1.4 (*n* = 14) versus −68.9±2.4 mV (*n* = 16, *p*<0.05) in SST neurons. We obtained activation and inactivation time constants by fitting Na^+^ currents induced at 0 mV in both PV and SST outside-out patches. The activation time constants showed no significant difference (0.03±0.004 ms for PV, *n* = 20; 0.04±0.003 ms for SST, *n* = 20; *p* = 0.08). However, the time constant of decay phase in PV was slightly smaller than that in SST neurons (0.23±0.01 versus 0.27±0.01 ms; *p*<0.05).

These results show that channel densities at the axon are similar in PV and SST neurons but dramatically higher than those at the soma. In addition, the results indicate that gating properties of axonal Na^+^ channels differ in these neurons, with SST channels activated at more depolarizing *V*_m_ levels. Indeed, the threshold of AP recorded in proximal axonal blebs was −47.8±1.6 mV in PV (*n* = 7) and −43.4±1.0 mV in SST neurons (*n* = 11, *p*<0.05), suggesting that the distinct gating property of axonal Na^+^ channels determines AP threshold difference observed at the soma.

### Channel Subtype Distribution at the Axon

Distinct voltage-dependent properties of axonal Na^+^ channels may result from different distribution patterns of channel subtypes along the axon. We therefore performed immunostaining experiments to reveal the molecular identity of axonal channels. We tested the specificity of Na_V_1.1 antibody using various approaches. As shown in Figure S4A, the Na_V_1.1 band in Western blot disappeared in the presence of a blocking peptide. The immunosignal of Na_V_1.1 was also eliminated by the blocking peptide (Figure S5A and B). Double staining of Na_V_1.1 using two antibodies against different epitopes yielded similar patterns of Na_V_1.1 signals (Figure S6A). Importantly, we found Na_V_1.1 immunosignals at the AIS of PV cells in wild-type animals but no detectable signal in any PV-containing neurites in homozygous *Scn1a* knockout (Na_V_1.1^−/−^) mice (Figure S6B and C). For specificity testing of the Na_V_1.6 antibody, we employed immunostaining in *Scn8a* knockout (Na_V_1.6^−/−^) mice. No detectable Na_V_1.6 signal was observed in tissues obtained from Na_V_1.6^−/−^ mice (Figure S7A and B). Because Na_V_1.2 knockout is prenatally lethal, we examined the antibody specificity using blocking peptide and two different antibodies. The blocking peptide effectively abolished the Na_V_1.2 band in Western blot (Figure S4B) and tissue immunosignals (Figure S5C and D). Immunosignals produced by two antibodies against different epitopes overlapped well with each other (Figure S7C). With these results, we concluded that, under our experimental conditions (i.e., light fixation of the tissue), the antibodies against Na_V_1.1, Na_V_1.2, and Na_V_1.6 used in this study were able to identify their targets with high specificity and thus could be used in the following experiments.

**Figure 5:**
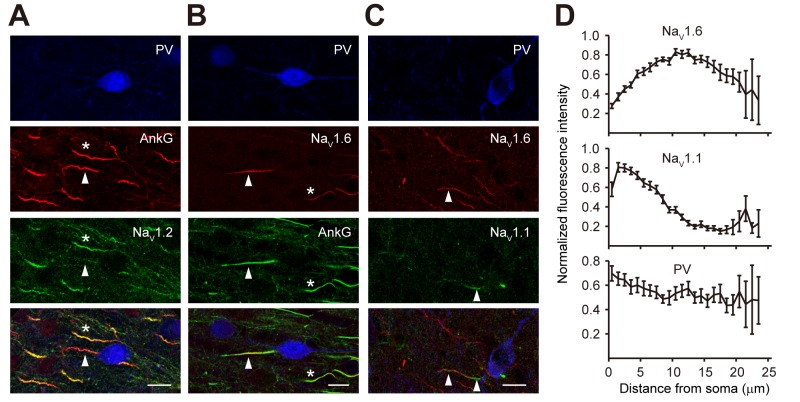
Polarized distribution of Na_V_1.1 and Na_V_1.6 at the AIS of PV neurons. (A) Triple staining using antibodies for PV (blue), AnkG (red), and Na_V_1.2 (green) revealed the absence of Na_V_1.2 at the AIS of PV neuron (arrowheads). Note that neighboring PV-negative AIS (presumably from PCs, asterisks) show strong immunosignals for Na_V_1.2. (B) Triple staining for PV, AnkG (green), and Na_V_1.6 (red). Note that distal regions of AIS were heavily stained for Na_V_1.6 (arrowheads). Neighboring axons (asterisks) also showed strong immunosignals. (C) Triple staining for PV, Na_V_1.6, and Na_V_1.1 shows polarized distribution of these subtypes at the AIS. (D) Plots of the averaged fluorescence intensity (± s.e.m., see Materials and Methods) as a function of distance from soma at the AIS. Data were obtained from triple-staining experiments similar to (C). Images are projections of confocal *z* stacks. Scale bars represent 10 µm. Error bars represent s.e.m.

As shown in Figure 5, we performed triple staining in PV neurons. Similar to the distribution profiles in PCs, Na_V_1.6 was found accumulated at the distal AIS regions of PV neurons (*n* = 37); however, Na_V_1.2 that accumulates at the proximal AIS of PC was found absent in all PV neurons examined (*n* = 58; Figure 5A and B). Instead Na_V_1.1 occupied the proximal AIS (Figure 5C). Similar to the segregated distribution of Na_V_1.2 and Na_V_1.6 at the AIS of PCs, selective distribution of proximal Na_V_1.1 and distal Na_V_1.6 along the AIS was observed in PV neurons (Figure 5C and D).

**Figure 6:**
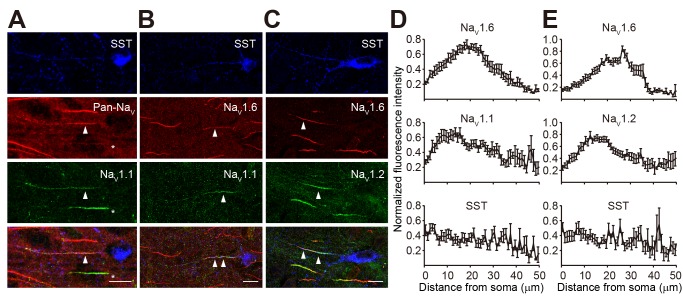
Polarized distribution of channel subtypes at the AIS of SST neurons. (A) Triple staining using antibodies for SST (blue), Pan-Na_V_ (red), and Na_V_1.1 (green) show modest intensity of Na_V_1.1 immunosignals at the AIS (arrowheads) and adjacent axon regions of SST neuron. Asterisks indicate a neighboring SST-negative axon (presumably PV axon) that was heavily stained. Nearby PC axons were not stained. (B) Triple staining for SST, Na_V_1.6 (red), and Na_V_1.1 (green) indicates co-localization of the two subtypes at the AIS. (C) Triple staining shows polarized distribution of Na_V_1.2 (proximal region) and Na_V_1.6 (distal region) at the AIS. (D and E) Plots of the averaged fluorescence intensity (± s.e.m.) as a function of distance from the soma. Data were obtained from triple-staining experiments similar to (B) and (C). Images are projections of confocal *z* stacks. Scale bars represent 10 µm. Error bars represent s.e.m.

For SST neurons, we also performed triple staining but used antibodies of pan-Na_V_, which recognizes all α subunits of Na^+^ channels, as the AIS marker (see Materials and Methods and Figure 6). The SST-labeled puncta outlined the structure of these cells (Figure 6A–C). The axons could be identified as strings of individual small puncta; they originated from the soma or dendrite and usually projected towards the pia. Ninety percent of SST neurons examined (*n* = 55/61) displayed immunosignals of Na_V_1.1 (Figure 6A and B), with higher intensity found at the proximal AIS (Figure 6D). In the remaining 10% of SST neurons, no Na_V_1.1 immunosignal could be detected, suggesting that these neurons may represent a distinct subpopulation of SST neurons. Interestingly, in all SST neurons examined, the proximal AIS showed strong fluorescence intensity of Na_V_1.2, whereas the distal AIS displayed intensive signals for Na_V_1.6 (Figure 6C and E). The distribution profile of Na_V_1.2 and Na_V_1.6 at the AIS of SST neurons was similar to that in PCs, with Na_V_1.2 accumulating at the proximal region of AIS and Na_V_1.6 concentrating at the distal AIS. In agreement with differences in estimated AP initiation sites (Figure 2), the length of AIS in SST was longer than that of PV neurons, and Na_V_1.6 immunosignals peaked at 20–30 µm from the soma in SST (Figure 6D and E), more distal than in PV neurons (10–15 µm; Figure 5D).

Immunostaining results show distinct distribution profiles of Na^+^ channel subtypes at the AIS of PV and SST neurons. In PV neurons, Na_V_1.1 and Na_V_1.6 accumulate at proximal and distal AIS, respectively, whereas Na_V_1.2 is completely absent from the AIS. In SST neurons, however, segregated proximal Na_V_1.2/Na_V_1.1 and distal Na_V_1.6 was observed; in addition, a more mixed distribution of high- and low-threshold channel subtypes was found at the AIS in the majority of SST neurons examined. Co-localization of high- and low-threshold channels in SST axons may result in a higher minimal activation voltage than that in PV axons.

### Contribution of Channel Subtypes to AP Threshold

Considering the differences in AIS length and channel subtype composition in PV and SST neurons, we performed simulations to identify the predominant factor that determines the difference in AP thresholds of these neurons. Because Na_V_1.1 and Na_V_1.2 are both the high-threshold subtype and the gating properties were similar, we used activation/inactivation curves of the PV soma to represent the Na_V_ subtype in soma. The low-threshold subtype was represented by activation/inactivation curves obtained from the PV AIS. In a simulation of outside-out patch, we inserted two subtypes of Na^+^ channels with gating properties similar to experimental observations (high threshold, nasoma; low threshold, naaxon). When the percentage of nasoma increased, the inactivation and activation curves were both right-shifted (Figure S8A), as indicated by depolarizing V_1/2_ values (Figure S8B). With a ratio close to 1∶1, the mixture of nasoma and naaxon yielded V_1/2_ similar to that found in outside-out patches excised from the AIS of SST neurons (−36.2 for activation and −70.1 mV for inactivation; Figure S8B).

We next performed simulations in a modeled neuron that had an axon with varying AIS length and channel subtype composition. The total number of Na^+^ channels were fixed but with varying ratios of nasoma to naaxon (Figure S8C, top). The AP threshold increased from −50.4 to −42.8 mV as the percentage of nasoma rose from 0% to 100% (Figure S8D, top). To examine the relationship between AIS length and AP threshold, we fixed the nasoma/naaxon ratio to 1∶1 but moved the location of peak channel density away from the soma and increased the overall AIS length (Figure S8D, bottom). The AP threshold showed a slight change from −48.2 to −48.7 mV when the peak density segment was relocated from 5 to 10 µm away from the soma. Together, these simulation results indicate that the level of subtype mixture instead of AIS length was the dominant factor in determining the AP threshold.

### Role of Na_V_1.2 in Regulating Network Activity

The presence of Na_V_1.2 in axons of inhibitory interneurons provides an explanation on why loss-of-function mutations of the *Scn2a* gene encoding Na_V_1.2 cause a genetic predisposition to epilepsy [25],[28],[29]. We next investigated whether a reduction of Na_V_1.2-mediated currents had an effect on the generation of recurrent network activity. Recent studies revealed that, at a low concentration, phrixotoxin-3 (PTx3) showed high selectivity in blocking Na_V_1.2 channels; tested on Na^+^ channel subtypes expressed in oocytes, the IC_50_ of PTx3 for Na_V_1.2 was a thousand-fold smaller than that for Na_V_1.1 [51],[52]. However, there is no result on its selectivity for native channel subtypes. Here we examined the role of PTx3 in regulating Na^+^ currents in different cell types (Figure S9). At a concentration of 30 nM (puff application), PTx3 showed no effect on Na^+^ currents in somatic nucleated patches of PV neurons (control, 232.4±64.8; PTx3, 229.7±73.0 pA, *n* = 6, *p* = 0.79; Figure S9A), but caused a significant reduction in those of SST neurons (244.5±65.4 versus 129.4±35.5 pA, *n* = 5, *p*<0.05; Figure S9B). A significant decrease was also observed in PC somatic Na^+^ currents (257.2±38.4 versus 97.8±16.7 pA, *n* = 5, *p*<0.01; Figure S9C) and those in outside-out patches from the proximal axon of PCs (220.0±53.8 versus 106.5±35.8 pA, *n* = 6, *p*<0.01; Figure S9D). In contrast, we found no significant change in distal axonal Na^+^ currents mediated by Na_V_1.6 channels (1.61±0.23 versus 1.48±0.34 nA, *n* = 5, *p* = 0.53; Figure S9E). Consistent with the immunostaining results showing the presence of Na_V_1.2 in both SST and PC but absence in PV neurons (Figures 5 and 6), these results indicate that PTx3 at a low concentration is a highly selective blocker for native Na_V_1.2 channels.

**Figure 7:**
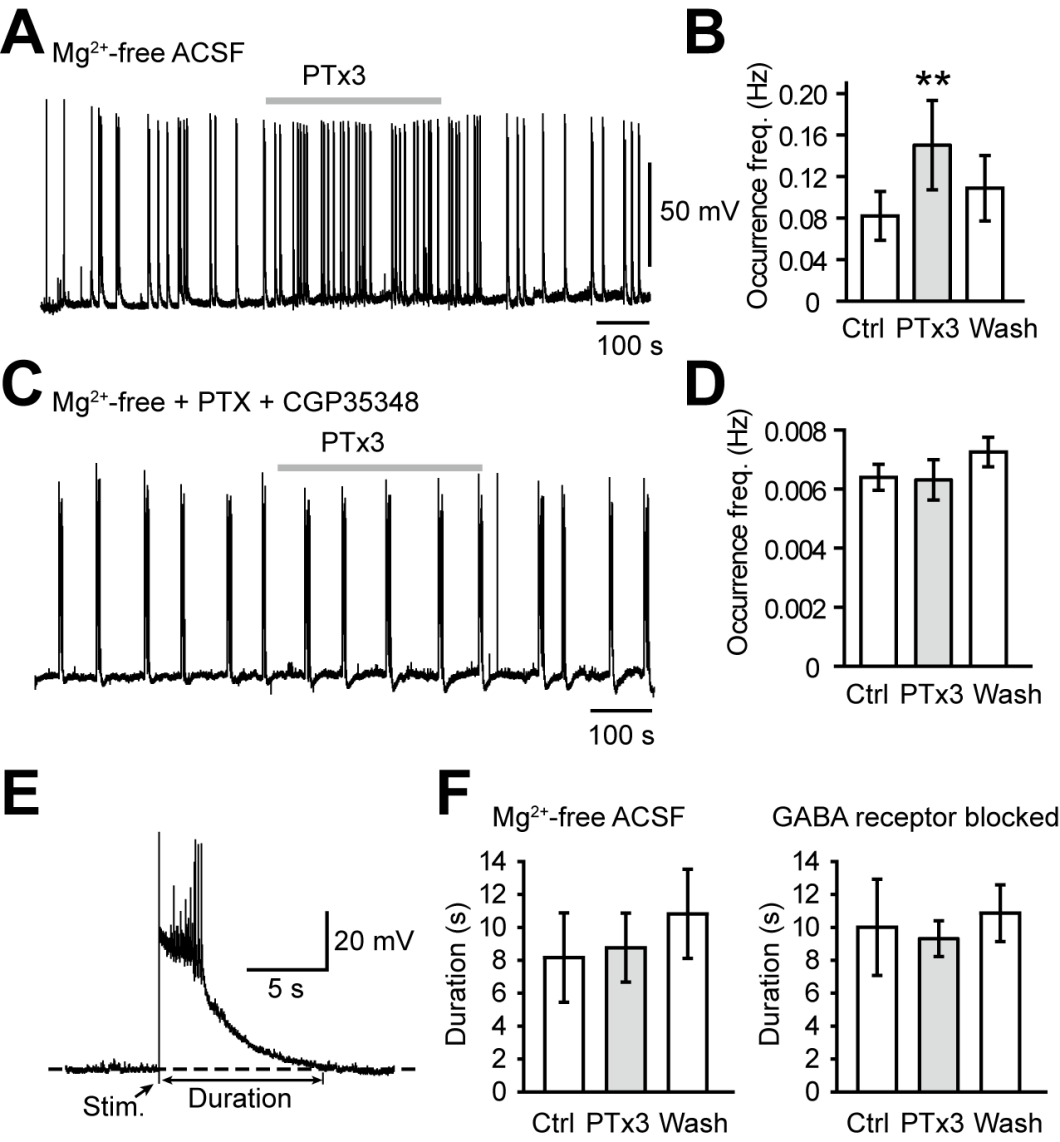
Reducing Na_V_1.2 currents promotes the generation of recurrent network activity. (A) Bath application of PaurTx3 (PTx3) increased the occurrence frequency of spontaneous network activity in a prefrontal cortical slice maintained in Mg^2+^-free ACSF (with GABA-mediated inhibition preserved). (B) Group data of Mg^2+^-free experiments (*n* = 6). (C) PTx3 showed no effect on spontaneous network activity in the presence of GABA receptor blockers (50 µM PTX and 100 µM CGP35348). (D) Group data of experiments using GABA receptor blockers (*n* = 7). (E) A network-activity event evoked by an electrical stimulation to the tissue showing the measurement of duration. (F) Group data showing that PTx3 had no effect on the duration of the network activity evoked in either conditions. For (B), (D), and (F), paired *t* test, ** *p*<0.01. Error bars represent s.e.m.

Next, we examined the effect of PTx3 on recurrent network activities in prefrontal cortical slices maintained in either Mg^2+^-free ACSF (Figure 7A and B) or with GABA receptors blocked (Figure 7C and D). In Mg^2+^-free ACSF, with GABA-mediated inhibition preserved, spontaneous network activity recorded from PCs was elevated by bath application of 30 nM PTx3, giving rise to an increase in the occurrence frequency (0.08±0.02 versus 0.15±0.04 Hz, *n* = 6 slices, *p*<0.05; Figure 7A and B). In contrast, also in Mg^2+^-free ACSF but with the presence of 50 µM PTX and 100 µM CGP35348, the occurrence frequency of network activity showed no significant change (0.007±0.001 versus 0.006±0.001 Hz, *n* = 7 slices, *p* = 0.31; Figure 7C and D). To investigate changes in the duration of network-activity events, we entrained the activity by delivering single electrical shocks to the slice (Figure 7E). Surprisingly, no change in the duration was observed after the application of PTx3 in either experimental condition (Figure 7F).

**Figure 8:**
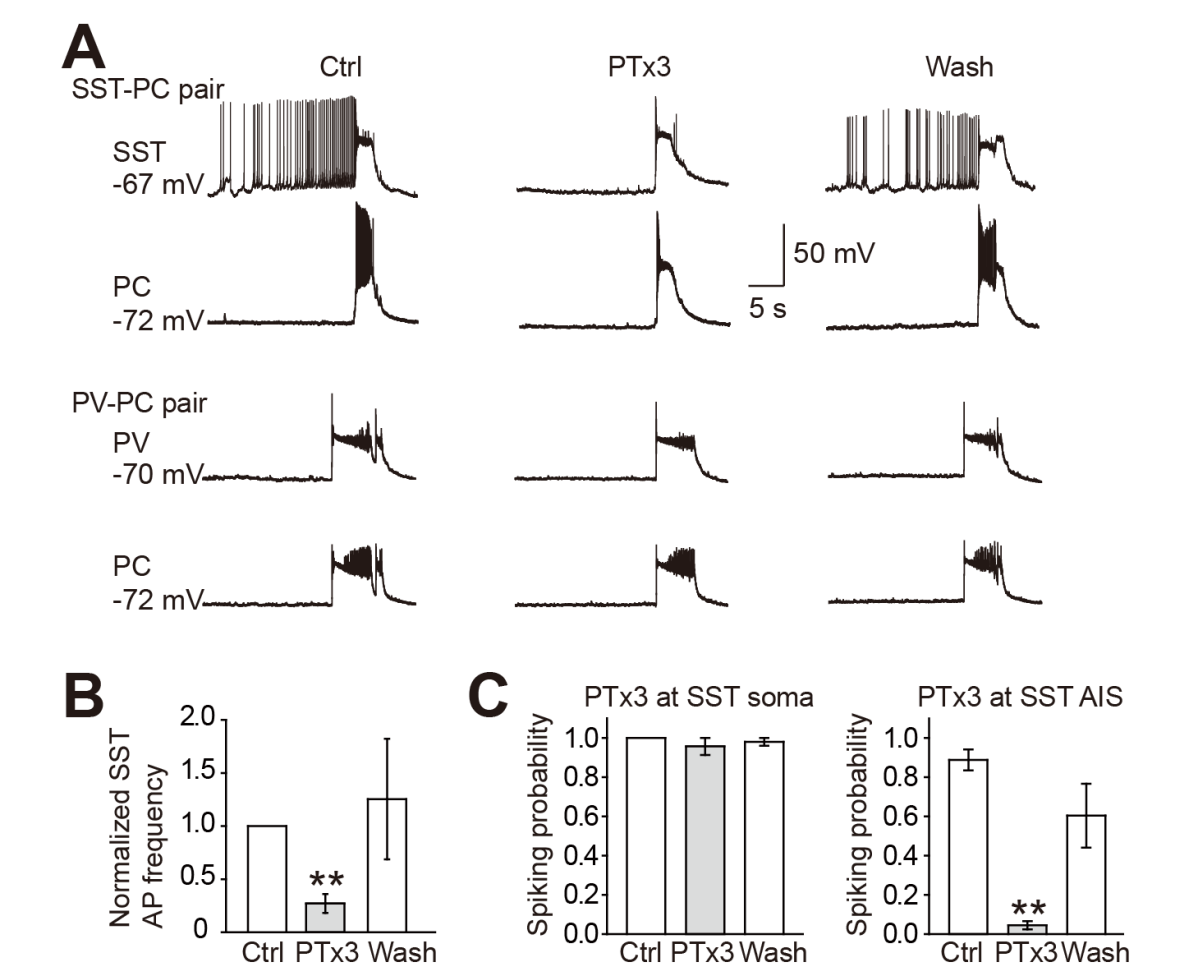
Spontaneous firing in SST neurons were suppressed by PTx3. (A) Example recordings from SST-PC and PV-PC pairs. PC and PV neurons showed no spontaneous activity during the refractory period between network-activity events; however, the SST neuron was constantly active. Spontaneous APs in the SST neuron could be substantially suppressed by bath application of 30 nM PTx3. (B) Group data showing that 30 nM PTx3 significantly decreased the frequency of spontaneous APs in SST neurons. (C) Puffing PTx3 (300 nM) at the soma had no effect on discharge probability in SST neurons (left), whereas puff at the AIS substantially decreased the firing probability (right). For (B) and (C), paired *t* test, ** *p*<0.01. Error bars represent s.e.m.

To further investigate the contribution of PC, PV, and SST neurons in the generation of recurrent network activity, we compared firing behavior of PCs and PV, and SST neurons in Mg^2+^-free ACSF. During the refractory period between network-activity events, PCs and PV neurons were usually silent; however, SST neurons were constantly active by generating spontaneous APs (Figure 8A). This result suggested a critical role of SST neurons in preventing the generation of epileptic events by providing incessant inhibition to the network. Those spontaneous activities in SST neurons were indeed inhibited by the bath application of PTx3. In the presence of 30 nM PTx3, the frequency of spontaneous APs was decreased to 27%±9% of control (Figure 8B). This decrease may result from the blockade of axonal Na^+^ channels in these neurons. Locally puffing PTx3 (300 nM) onto the soma showed no effect on the spiking probability (0.96±0.04, *n* = 3, *p* = 0.42); however, puffing onto the proximal axon abolished AP generation in SST neurons (control, 0.89±0.05; PTx3, 0.05±0.02, *n* = 6, *p*<0.001; Figure 8C).

In agreement with previous reports showing various inheritable epileptic syndromes in human patients with loss-of-function mutations in Na_V_1.2 [25],[28],[29], these results indicate that a global reduction in Na_V_1.2-mediated currents could promote the initiation but not the maintenance of recurrent network activity. Spontaneous activities in SST neurons during the refractory period may provide tonic inhibition to the apical dendrites of principal cells to prevent their burst firing and thus the initiation of recurrent network activity.

## Discussion

In this study, we demonstrate that the gating properties of axonal Na^+^ channels vary across interneuron subtypes. The difference was found at the AIS, a structure usually thought to be conservative in its channel composition, giving a new perspective on interneuron diversity. The low minimal activation voltage of Na^+^ channels in the AIS presumably confers PV neurons with greater responsiveness to stimuli by lowering the AP threshold. In SST neurons, Na^+^ channels less susceptible to inactivation maintain a stable firing ability at different *V*_m_ levels. This variation in gating properties of AIS Na^+^ channels raised from considerable segregation of high- and low-threshold channel subtypes in PV neurons. All three subtypes of Na^+^ channels in the cortex (i.e., Na_V_1.1, Na_V_1.2, and Na_V_1.6) were expressed in the AIS of SST neurons showing a more intermingled distribution pattern than in PV neurons. Therefore, our results demonstrate that the diversity of inhibitory interneurons extends to the axonal level; specific distribution of various Na^+^ channel subtypes at the AIS gives rise to diversity in interneuron excitability and serves as a critical target for the regulation of excitation-inhibition balance in the cortex.

Previous findings demonstrated that APs are preferably generated in the axon of SST-positive neurons in the hippocampus, but the dendrite can also generate APs in response to brief stimulations [45]. In our study, we determined the site of AP initiation by simultaneous recordings from the soma and axonal blebs of neocortical PV and SST neurons [1]. Similar to the case in PCs [1],[2], the AP initiation sites in the two types of interneurons were found at the AIS. But the position of the AP initiation site in PV neurons was generally localized closer to the soma (Figure 2), which likely promotes fast activation of PV neurons. Previous studies suggested that densely distributed Na^+^ channels at the AIS promote the generation of APs in PCs [6]–[8],[53]; however, it remains unknown whether this also applies to inhibitory interneurons. By taking advantage of patch recording from axonal blebs [1],[16],[46], we demonstrate that Na^+^ channel density at axonal patches excised at or near the AIS is 50–60 times higher than that at somatic patches from interneurons (Figure 4). This ratio was slightly higher than that found in PCs [10],[54]. In agreement with these results, immunostaining also revealed a high channel density at the AIS of PV and SST cells (Figures 5 and 6). A recent study in hippocampal PV neurons demonstrated that a high density of axonal Na^+^ channels is required for their high-frequency repetitive firing and fast AP propagation [55].

Most of the current knowledge regarding properties of the axonal Na^+^ channel comes from studies on PCs. Electrophysiological characteristics of Na^+^ channels in interneurons have not received much attention, possibly due to difficulties in patch recording from interneuron axons. Previous findings have demonstrated similar voltage dependence of somatic Na^+^ channels in hippocampal PCs and basket cells, both of which express high-threshold Na^+^ channels at the soma [50]. In agreement with these findings, we found that somatic Na^+^ currents obtained from nucleated patches showed a high minimal activation voltage in both PV and SST neurons. However, Na^+^ currents recorded from axonal patches of PV neurons activated at a considerably lower *V*_m_ level (∼7 mV more hyperpolarized) than that of SST neurons. A hyperpolarizing shift of the activation curve enables PV neurons to initiate APs at substantially lower *V*_m_ levels. Indeed, the AP threshold in PV was ∼7 mV lower than in SST neurons. SST neurons are well-known for their low rheobase, which is somewhat paradoxical considering their relatively high AP threshold. Considering that SST neurons receive facilitatory EPSPs and fire APs during late phases of the sustained stimulation (i.e., with long onset latency of APs) [40], higher AP threshold would meet the needs for preventing AP generation during early phases of stimulation. However, more depolarized resting *V*_m_, high input resistance, and low rheobase may increase the probability of firing after receiving prolonged excitatory inputs. In addition, the small after-hyperpolarization may enable sustained firing when the summated EPSPs reach a level above the AP threshold. Depolarized resting *V*_m_ in SST neurons would also inactivate a large fraction of Na^+^ channels if their channels have similarly low minimal inactivation voltage to those of PV neurons. However, SST neurons keep their excitability by positively shifting the minimal inactivation voltage of Na^+^ channels, and indeed AP threshold in SST neurons is less susceptible to *V*_m_ fluctuation (Figure 1). Other channels such as K^+^ and Ca^2+^ channels also play important roles in determining neuronal excitability and AP waveforms. During AP generation, Na^+^ channels and K^+^ channels function as the “engine” and “brake” of neuronal signaling, in which K^+^ channels activate later to counter the depolarization induced by opening of Na^+^ channels. In our stimulation paradigm using 2-ms current injection, APs are generated too quickly for K_V_1 channels to gain their full strength to exert the “dampening” effect that elevates the AP threshold. Thus, in response to brief but strong stimuli, the AP threshold is much less susceptible to K_V_1 channel blocker α-DTx than it is when prolonged but weak stimuli are applied. However, considering that Na^+^ channels are the most important player for AP initiation, we focused on Na^+^ channels in this study.

Interestingly, when the *V*_m_ was maintained at −50 mV by DC current injection, AP thresholds in PV and SST neurons were similar, presumably resulting from inactivation of low-threshold AIS Na^+^ channels (i.e., Na_V_1.6). These results indicate that in terms of channel readiness PV neurons had a higher firing capability at resting state but AP firing was more susceptible to *V*_m_ changes. On the other hand, firing capability of SST neurons remained relatively stable across various *V*_m_ levels. These observations correspond well with the differential activity patterns of PV and SST neurons. In response to a stimulus train, PV neurons discharge immediately, then remain silent through the period of stimulation; on the contrary, SST neurons are not initially activated, but they fire consistently in the late phase of stimulation [40]. This “routing” phenomenon has been attributed to differential synaptic integration in excitatory synapses onto PV and SST neurons [41]–[43]. The expression of voltage-gated Na^+^ channels in interneuron dendrites also contributes to this phenomenon [44],[45]. The relatively high density of Na^+^ channels in SST dendrites may promote EPSP synchronization and cause the generation of multiple APs in the later phase of the stimulus train. Our results suggest that the voltage dependence of AIS Na^+^ channels also plays an important part in determining these distinct firing behaviors in different interneuron types. In this study, we focused on the two predominant interneuron types, the PV and SST neurons, which constitute approximately 39% and 23% of the total population of inhibitory interneurons [36]; the properties of axonal Na^+^ channels in other types of inhibitory interneurons, such as those containing CCK, Calbindin, and Calretinin, remain to be further examined.

The difference in activation of axonal Na^+^ channels may reflect cell-specific composition and distribution of Na^+^ channel subtypes along the axons of these interneurons. Using triple immunostaining we revealed the molecular identity of axonal Na^+^ channels of PV and SST neurons. In agreement with previous studies, Na_V_1.1 was found accumulated at the proximal portion of the AIS in PV neurons, whereas the distal AIS was populated by Na_V_1.6 [18],[19]. Interestingly, all three channel subtypes identified in the cortex are expressed at the AIS of SST neurons: the peak immunosignals of Na_V_1.1 and Na_V_1.2 were localized closer to the soma, whereas Na_V_1.6 signals peaked at a more distant location at the AIS. Although the segregated distribution of high-threshold (Na_V_1.1 in PV, or Na_V_1.1 and Na_V_1.2 in SST) and low-threshold subtypes (Na_V_1.6) was observed in both PV and SST neurons, the degree of segregation was greater in PV neurons. The presence of high-threshold channels along the entire AIS may result in higher voltage threshold for axonal Na^+^ channels and AP generation in SST neurons.

Previous studies indicated that Na_V_1.1 was specifically expressed in cortical interneurons, and Na_V_1.2 was only expressed in excitatory PCs [10],[18],[19]. Here we demonstrate that Na^+^ channel subtype expression in the axon varies between interneuron subtypes. Importantly, we revealed the expression of Na_V_1.2 at the AIS in all SST neurons examined. Together with the finding that PCs express Na_V_1.2, our results suggest that regulation of Na_V_1.2 expression or activation may cause changes in both excitation and inhibition, which may disrupt the excitation-inhibition balance in the neocortex.

Because Na_V_1.1 was found to be specifically expressed in GABAergic neurons, most studies on Na^+^ channel pathology in interneurons focused on contribution of Na_V_1.1 to epileptogenesis [21]–[24]. Loss-of-function of Na_V_1.1 causes disinhibition in the cortex, which in turn results in hyperexcitability of the cortical network and the development of epilepsy symptoms [24]. In contrast, the role of mutated Na_V_1.2 as an epilepsy ethiological factor has long been debated. It has been shown that gain-of-function of Na_V_1.2 led to benign familial neonatal-infantile seizures [26],[27]; however, loss-of-function of Na_V_1.2 was also identified as a promoter of epilepsy [25],[28],[29]. Based on the evidence that Na_V_1.2 channels are expressed in inhibitory SST neurons as well as in PCs (Figures 5 and 6), we speculated that regulating activities of Na_V_1.2 may shape network activity. Indeed, the application of Na_V_1.2 blocker PTx3 [51],[52] considerably increased the occurrence frequency of spontaneous synchronized network activities in slices perfused with Mg^2+^-free ACSF. Considering that both PC and SST neurons express Na_V_1.2, we performed recordings form slices maintained in Mg^2+^-free ACSF but with GABAergic inhibition eliminated from the network. The occurrence frequency of synchronized network activities remained unchanged after the application of PTx3, suggesting a critical role of SST neurons in regulating the generation of recurrent network activity.

In response to global reduction of Na_V_1.2 channel activity, the loss of inhibition from SST neurons overweighed the loss of excitation from PCs, leading to a reduction in the threshold for the generation of recurrent network activity. This notion is supported by the observation that SST neurons discharge spontaneous APs. Constant firing of SST-positive LTS neurons was also described during the up-down state, suggesting this firing mode is an inherent mechanism to keep cortical excitation-inhibition balance during active brain states [56]. Loss of Na_V_1.2 reduces firing probability of SST neurons and thus silences a sustained source of cortical inhibition. SST neurons mainly send their axons to innervate the apical dendrites of PCs and provide inhibition to suppress burst firing of these principal cells, thus leading to a reduction in probability of initiating network activities including epileptiform activity. In an interesting similarity with Na_V_1.2, gain-of-function and loss-of-function mutations of Na_V_1.6 have both been associated with different forms of epilepsy [31],[32],[57]. Considering that both subtypes are distributed in the axon of interneurons as well as PCs, the underlying mechanism of loss-of-function mutations could be the imbalance of cortical excitation and inhibition caused by decreased output of interneurons.

In conclusion, the expression of axonal Na^+^ channel subtypes varies in different types of interneurons; distinct subtype combinations at the AIS determine the neuronal excitability. The lower minimal activation voltage of AIS Na^+^ channels grants PV neurons higher discharge probability at resting states, whereas the higher threshold of the AIS Na^+^ channels in SST neurons ensures the firing capability of SST neurons is more resistant to *V*_m_ depolarizations. Importantly, the distribution of Na_V_1.2 and spontaneous firing in SST neurons during the refractory period highlights the role of SST neurons in preventing the initiation of recurrent network activity.

## Materials and Methods

### Ethics Statement

The use and care of animals complied with the guidelines of the Animal Advisory Committee at the Shanghai Institutes for Biological Sciences.

### Slice Preparation

Coronal slices of prefrontal cortex were prepared from P16–22 PV-GFP transgenic mice (B13) and GAD-GFP transgenic mice (GIN) with SST-expressing neurons predominantly labeled [47],[48]. We anesthetized the mice with intraperitoneal (i.p.) injection of sodium pentobarbital (100 mg per kg of body weight). Animals were then killed by decapitation, and brain tissues were immediately dissected out and immersed in ice-cold oxygenated (95% O_2_ and 5% CO_2_) slicing solution. The composition of this solution was similar to that of normal ACSF (described below) except that NaCl was replaced by equiosmolar sucrose. Slices (250 µm in thickness) were cut with a Leica microtome (VT-1000S) and immediately transferred to an incubation beaker filled with aerated normal ACSF containing (in mM): NaCl 126, KCl 2.5, MgSO_4_ 2, CaCl_2_ 2, NaHCO_3_ 26, NaH_2_PO_4_ 1.25, and dextrose 25 (315 mOsm, pH 7.4, 35°C). After at least 45 min of incubation, we transferred slices to a submerged chamber perfused with aerated ACSF and visualized cortical neurons with an upright infrared differential interference contrast microscope (BX51WI, Olympus). Experiments investigating the voltage dependence and time constants of Na^+^ currents were carried out at room temperature (∼25°C), and all other recordings were performed at 36–36.5°C.

### Electrophysiological Recordings

Direct recording of axonal cut-ends (blebs) enabled us to study Na^+^ channels in the AIS and axonal trunk [1],[10],[16],[46],[58]. We performed patch-clamp and whole-cell recordings from layer 2–5 interneurons designated by fluorescence with a Multiclamp 700B amplifier (Molecular Devices). For patch-clamp experiments we used pipettes of similar resistance (7–10 MΩ) filled with (in mM): CsCl 145, MgCl_2_ 2, Na_2_ATP 2, HEPES 10, EGTA 0.2, and TEA (tetraethylammonium) 2 (286 mOsm, pH 7.2 with CsOH). We added biocytin (0.2%) to the pipette solution for post hoc DAB staining in order to trace and measure the axon length of recorded neurons. To isolate the Na^+^ currents, we added 4-AP (4-Aminopyridine, 3 mM) and CdCl_2_ (100 µM) to the bath solution to block voltage-gated K^+^ and Ca^2+^ channels, respectively. To measure and compare the channel density at the soma and the axon, we performed recordings from outside-out patches excised from the soma and the axonal bleb using patch pipettes with similar taper shape and impedance. We obtained nucleated patches from the soma and outside-out patches from the axonal blebs to investigate the difference in voltage dependence between somatic and axonal Na^+^ currents. In some experiments, we performed recordings from isolated axonal blebs of PCs. To isolate axonal blebs from the main axon trunk, we made a cut at the border between layer 6 and the white matter by inserting a sharp electrode into the tissue and then sweeping the electrode along the border [10],[58].

We recorded families of Na^+^ currents evoked by consecutive depolarizing voltage steps from −80 mV to +40 mV (10 mV per step; duration, 30 ms) following a prepulse of −120 mV (duration, 50 ms) and generated the activation curves based on the peak currents at each step. Minimal activation voltage of the Na^+^ currents was defined as the voltage at which the peak current reached 10% of its maximum value. To obtain the inactivation curves, we plotted the peak currents evoked by a test pulse (30 ms) to 0 mV following a range of prepulses (50 ms) from −120 to −30 mV. Currents were filtered at 10 kHz and sampled at 50 kHz using a Digidata 1440A interface and pClamp10 software (Molecular Devices). Current traces underwent an on-line digital subtraction of leakage currents using a P/4 procedure, in which the currents evoked by four hyperpolarizing pulses with one-fourth amplitude of the test pulse P were summed and added to the current trace evoked at pulse P. Activation and inactivation data were fitted with Boltzmann function. Current traces were fitted with Hodgkin-Huxley function to obtain the activation time constant. The time constant of decay was obtained from a single exponential fit. The current density of a somatic nucleated patch was determined by dividing the peak current evoked during the activation protocols by membrane area calculated from the diameter of the nucleated patch. Conductance density of nucleated patches was computed as previously described [59].

In experiments determining the AP thresholds, we made whole-cell recordings from the soma using pipettes filled with K^+^-based internal solution containing (in mM): K gluconate 140, KCl 3, MgCl_2_ 2, Na_2_ATP 2, HEPES 10, and EGTA 0.2 (288 mOsm, pH 7.2 with KOH). To characterize the firing patterns of PV and SST neurons, current stimulation with a duration of 500 ms was applied to the soma. In some experiments, bath application of α-DTx (100 nM) was used to reveal the contribution of K_V_1 channels to a delay-type firing pattern. APs evoked by brief somatic current steps (duration, 2 ms) at an interval of 2 s were used for threshold measurement. Unless otherwise stated, the AP threshold was determined as the *V*_m_ level at which the derivative of the voltage (d*V*/dt) equals 20 V/s. AP width was determined as the duration at half AP amplitude that was measured from AP threshold to peak. For estimation of AP initiation sites, we performed simultaneous whole-cell recording from the soma and the axonal bleb. Somatic and axonal APs were evoked by brief current injections (duration, 2 ms) at either the soma or the axonal bleb. Based on an assumption that APs propagate along the axon at the same velocity irrespective of traveling direction, we established a group of equations: 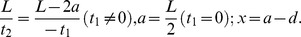, where *x* is the estimated distance between the soma (axon branching point) and the AP initiation site, *L* is the axon length between the center of soma and the axonal recording site, *a* is the distance between the center of soma and the initiation site, *d* is the radius of the soma, and *t*_1_ (or *t*_2_) is the time difference between peaks of axonal and somatic APs evoked by 2-ms current pulse at the soma (or axon). The time *t*_1_ and *t*_2_ were calculated as: somatic – axonal AP peak time. Because somatic stimulation will cause AP initiation at the regular initiation site and then propagation to the axonal recording site and backpropagation to the somatic recording site, *t*_1_ is actually the time spent on the distance *L* – 2*a* (for *t*_1_<0; that is, somatic AP occurs earlier than axonal AP) or 2*a* – *L* (for *t*_1_>0; that is, axonal AP occurs earlier). When AP arrives at somatic and axonal recording sites simultaneously—that is, *t*_1_ = 0—the estimated initiation site locates at the midpoint of *L*. In whole-cell current clamp experiments, the voltage signals were sampled at 50 kHz using a CED Micro1401 and Spike 2 software. Recordings with an access resistance larger than 20 MΩ at the soma and 25 MΩ at the axon were discarded. Liquid junction potentials were not corrected for *V*_m_ values shown in the text and figures.

### Generation of Recurrent Network Activity in Vitro

We maintained prefrontal cortical slices in Mg^2+^-free ACSF (3.5 mM K^+^) or with GABA_A_ and GABA_B_ receptor blockers (50 µM picrotoxin and 100 µM CGP35348) added to this Mg^2+^-free ACSF. Spontaneous recurrent network activities could be detected under these conditions. After stable occurrence of recurrent network activities were observed, we applied 30 nM phrixotoxin-3 (PTx3 for short), a selective Na_V_1.2 blocker, to examine its effect on the generation of the spontaneous activities. The time period during PTx3 application was approximately 5 min, and the toxin was then washed out with ACSF used in control. To examine the effect of PTx3 on the duration of network-activity events, we delivered extracellular stimulation (150–300 µA, 100 µs) to the slice to evoke and entrain recurrent network activities. The duration of these evoked activities was determined as the time difference between the stimulation artifact and the time point when *V*_m_ dropped to the baseline (mean value of *V*_m_ during the 1-s period before the artifact). After achieving simultaneous recording from PC-SST pairs, the number of spontaneous APs during the refractory period (between recurrent network events) was obtained and compared to that with the treatment of PTx3. In experiments examining the effect of PTx3 on the capability of AP generation, 300 nM PTx3 was puffed onto the soma or the proximal portion of the axon while monitoring the probability of AP generation in response to 2-ms current injections. Previous findings in PCs suggested that somatic and distal axonal Na^+^ currents are mediated by Na_V_1.2 and Na_V_1.6 channels, respectively. To determine the specificity of PTx3 on these channel subtypes, we examined the effects of PTx3 on Na^+^ currents obtained from nucleated patches, outside-out patches from the proximal AIS, and isolated axonal blebs of PCs. We performed similar pharmacological experiments in somatic nucleated patches excised from PV and SST neurons. PTx3 (30 nM) was puffed to nucleated patches or isolated blebs using patch pipettes with large opening tips.

### Purification of Na_V_1.1 Blocking Peptide

The sequence that encoded 1918–1998 aa of Na_V_.1.1 (NCBI Reference Sequence: NM_018733.2) was cloned from mouse cortical cDNA and inserted into pET-28a vector. The construct was then transformed to *E. coli*. We grew the culture containing the ΔNa_V_1.1-His-tagged fusion protein to an O.D.600 between 0.4 and 0.6 and then induced protein expression. For IPTG induction, we added IPTG to a final concentration of 1 mM and incubated it at 30°C for 5 h. The bacteria were then harvested and sonicated. The ΔNa_V_1.1-His-tagged fusion protein was purified by using Ni-NTA magnetic beads according to the Promega protocol.

### Immunoblotting

The tissue samples were homogenized in 1∶10 (w/v) ice-cold radioimmunoprecipitation assay (RIPA) buffer [in mM, 150 NaCl, 5 EDTA, 1% Triton X-100, 1 Na3VO4, 50 NaF, 1 PMSF, 1 aprotinin, 1 leupeptin, 5 DTT, protease inhibitor cocktail (Sigma-Aldrich P8340), and 10 Tris-Cl, pH 7.4]. The homogenates were centrifuged at 13,000 *g* for 15 min at 4°C. Supernatants were collected and added to 4× SDS sample buffer. Samples were stored at −20°C until assay and were thawed only once. Mice cortical extracts (100 µg) were electrophoresed on SDS/5% PAGE and transferred to polyvinyldifluoridine membranes. The membranes were incubated overnight at 4°C with the primary antibodies Na_V_1.1 (73-023, 1∶200, NeuroMab) and mouse anti Na_V_1.2 (73-024,1∶500, NeuroMab) followed by the horseradish peroxidase–conjugated anti-mouse secondary antibody (Amersham Biosciences) 2 h at room temperature. The protein bands were visualized using the Pierce ECL system and scanned. The concentrations of blocking peptides were 1∶20 for Na_V_1.1 and 1∶1 for Na_V_1.2.

### Immunostaining

We investigated the distribution patterns of three Na^+^ channel subtypes (Na_V_1.1, Na_V_1.2, andNa_V_1.6) that dominate in the mature central nervous system. Antibodies for PV and SST or for GFP were used to identify the cell subtypes. The AIS could be identified using AnkyrinG (AnkG) and pan-Na_V_ antibodies.

The *Scn1a* knockout (Na_V_1.1^−/−^) and *Scn8a* knockout (Na_V_1.6^−/−^) mice were used to test antibody specificity and genotyped as previously described [57],[60]. Homozygous KO mice and their wild-type littermates were selected for the experiments. C57/B6 mice (P16–P22) were deeply anaesthetized with sodium pentobarbital (i.p.) and then perfused through the hearts with normal saline (12–15 ml) followed by ice-cold fixative (8–10 ml). The brain was dissected out and postfixed in the same fixative for about 2 h. For triple staining using PV, AnkG, and Na_V_1.6 antibodies, the fixative contained 2%–3% paraformaldehyde (PFA) and 2%–3% sucrose (in 0.1 M phosphate buffer, PB, pH 7.4). For other stainings, we used fixative containing 1% PFA and 1% sucrose. In some experiments, PFA and sucrose with even lower concentrations (0.5%) were used for Na_V_1.2 staining. After postfixation, the brain tissues were immersed in 30% sucrose in 0.1 M PB overnight. We obtained cryostat coronal sections (16 µm in thickness) using a freezing microtome.

We rinsed the sections in 0.01 M phosphate-buffered saline (PBS, pH 7.4) 3 times and incubated them in 0.5% Triton X-100 (in PBS) for 0.5 h and then in blocking solution (10% BSA in PBS) for 1 h. Sections were incubated overnight at room temperature with the primary antibodies in 0.2% Triton. Antibodies used in this study were: goat anti-AnkG (sc-31778, 1∶400, Santa Cruz), mouse anti-AnkG (sc-12719, 1∶400, Santa Cruz), Pan-Na_V_ (ASC-003, 1∶500, Alomone Labs), mouse anti-Na_V_1.1 (73-023, 1∶200, NeuroMab), rabbit anti-Na_V_1.1 (AB5204, 1∶100, Millipore), mouse anti-Na_V_1.2 (73-024,1∶200, NeuroMab), rabbit anti-Na_V_1.2 (ASC-002, 1∶400, Alomone), rabbit anti-Na_V_1.6 (ASC-009, 1∶500, Alomone Labs), goat anti-PV (PVG-214, 1∶1,000, Swant), mouse anti-PV (MAB1572, 1∶1,000, Millipore), rabbit anti-PV (PV25, 1∶1,000, Swant; PV28, 1∶3000, Swant), and goat anti-SST (sc-7819, 1∶200, Santa Cruz). After a complete wash in PBS, sections were incubated in the following secondary antibodies (1∶1,000; Invitrogen) for 2 h: Alexa 488–conjugated donkey anti-rabbit, Alexa 555–conjugated donkey anti-mouse, and Alexa 647–conjugated donkey anti-goat. Sections were then mounted on slides with fluoromount-G (Electron Microscopy Science). We took images with a laser scanning confocal microscope (Nikon FN1) with a 60× objective and an appropriate zoom. Automated sequential acquisition of multiple channels was used to obtain single or *z*-stack images with an interval of 0.5 or 1 µm.

For quantitative analysis of fluorescence intensity along the AIS, we extracted the fluorescence signals only at the AIS with the FIJI software and calculated the fluorescence intensity in three dimensions using the Amira software. Deconvolved by Autoquant X2 software (Media Cybernetics), all three channels of fluorescence signals were integrated in an 8-bit image with image expression parser function. We traced the AIS in the image, filled it out, and extracted the mask image of this fillout. A combination of the fillout mask and deconvolved fluorescence images of three channels produced an image with fluorescence signals only present at the AIS. We then calculated the fluorescence intensity along the AIS using the Amira software. We averaged fluorescence signals every 1 µm. After normalization to the maximum fluorescence intensity in each AIS, we plotted the averaged relative fluorescence intensity against the distance from the soma using Matlab.

### Model Construction

We employed two voltage-gated Na^+^ channels, nasoma and naaxon, to simulate channels at the soma and the distal AIS. Their dynamics were governed by Hodgkin-Huxley–style equations. The voltage dependence parameters of nasoma and naaxon were initially set to values observed in experiments (Figures 3 and 4). In a single compartment model (length, 2 µm; diameter, 2 µm), we inserted nasoma and naaxon (20 pS/µm^2^) with various ratios to test voltage-dependent properties of mixed channel subtypes.

A computational model of a simplified neuron was implemented with NEURON 7.3 simulation environment, containing an oval soma (length, 20 µm; maximal diameter, 15 µm), axon hillock (length, 1 µm; diameter tapered from 2 to 0.7 µm), and a segment of cylindrical AIS (length, 21–26 µm; diameter, 0.5 µm). The electrical properties Rm, Cm, and Ri were set to 10,000 Ω·cm^2^, 0.2 µF/cm^2^, and 50 Ω·cm, with Cm of soma set to 1 µF/cm^2^. The resting *V*_m_ at the soma was set to −70 mV. All simulations were run with 10-µs time steps, and the nominal temperature of simulation was 37°C.

Transient Na^+^ currents were present throughout the model cell. The distribution of channel subtypes was set according to their profiles observed in experiments. Specifically, nasoma was present in soma, hillock, and proximal AIS, whereas naaxon was present at the proximal AIS but dominant at the distal AIS. Channel density at the soma was fixed to 25 pS/µm^2^. The peak density (5-µm-long) at the AIS was maintained at 1,000 pS/µm^2^, but the ratio of nasoma to naaxon was set to different values. The Na^+^ equilibrium potential was set to 60 mV.

The fast voltage-gated K^+^ current was present at the soma (25 pS/µm^2^), hillock, and AIS (1,000 pS/µm^2^). The density of slow noninactivating K^+^ current (M current) was set to 5 pS/µm^2^ at the soma and 25 pS/µm^2^ at the hillock and the AIS. The K^+^ equilibrium potential was set to −90 mV. Activation and inactivation curves were fitted with Boltzmann functions. APs evoked by brief somatic current steps (duration, 2 ms) were used for threshold measurement.

### Statistical Analysis

All numerical values in the text and figures are presented as mean ± s.e.m. Statistical tests were performed using Student's *t* test or ANOVA.

## Supporting Information

Figure S1 **Blocking K_V_1 does not change the threshold of APs evoked by brief and strong stimulation.** (A, Left) *V*_m_ responses of a PV neuron to 250-pA (threshold current) and 400-pA step current injections. (Right) The same neuron after application of 100 nM α-DTx. The threshold current decreased to 100 pA, and the delay of the first AP (indicated by the gray bar) was diminished. (B, Left) *V*_m_ responses of a SST neuron to 10-pA (threshold current) and 200-pA step current injections. (Right) The same neuron after application of 100 nM α-DTx. The duration of the depolarizating ramp before the first AP was not affected. (C, Left) Averaged delay of the first AP in both neuronal types before and after application of 100 nM α-DTx. Delay in SST neruons is the duration of the depolarizing ramp before the first AP. (Right) Voltage threshold changes of the first AP in both neuronal types before and after application of 100 nM α-DTx. (D) Voltage threshold changes of single APs induced by 2-ms current pulses in both neuronal types before and after α-DTx application. No significant difference was found between the control and α-DTx groups. *** *p*<0.001; ** *p*<0.01. Error bars represent s.e.m.
(TIF)

Figure S2 **Soma- and dendrite-originated axons in PV and SST neurons.** (A) PV neurons with axons originated from the soma (left) or the dendrite (right). (B) SST neurons. Arrowheads indicate the axonal blebs. The majority of PV and SST neurons emit their axons from the soma (86.7% of PV and 80.6% of SST neurons), whereas the remaining cells emit axons from dendrites.
(TIF)

Figure S3 **AIS Na^+^ channels determine the lowest AP threshold.** (A) Example recording from a PV neuron showing the effect of perisomatic application of TTX (10 µM, puffing) on AP threshold and waveform. (Top) Schematic diagram showing the location of TTX application (gray area). (B) Perisomatic TTX showed no effect on AP thresholds but caused a dramatic decrease in peak amplitudes of APs. (Left) Three APs from (A) (arrowheads). (Right) Phase plot of APs. (C) Group data showing changes in AP threshold and peak amplitude of d*V*/dt. (D–F) Similar recording and analysis as in (A–C) except that TTX was applied at the AIS. TTX dramatically increased the AP thresholds but had no change in peak d*V*/dt. (G) In SST neurons, perisomatic TTX substantially decreased the peak amplitude of APs but not the threshold. (H) TTX application at the AIS of SST neurons significantly increased the AP thresholds. For statistical data, ** *p*<0.01. Error bars represent s.e.m.
(TIF)

Figure S4 **Verification of Na_V_1.1 and Na_V_1.2 antibodies with Western blot.** Western blot analysis of the cortical extracts from C57 mice using (A) Na_V_1.1 and (B) Na_V_1.2 antibodies with or without pre-incubation of antigenic peptides.
(TIF)

Figure S5 **Immunosignal of Na_V_1.1 and Na_V_1.2 was eliminated by blocking peptides.** (A) Double staining of AnkG and Na_V_1.1. (B) Double staining of AnkG and Na_V_1.1 in the presence of blocking peptide. (C) Double staining of AnkG and Na_V_1.2. (D) Double staining of AnkG and Na_V_1.2 in the presence of blocking peptide. Scale bar in (A–B), 50 µm; scale bar in (C–D), 10 µm.
(TIF)

Figure S6 **Verification of Na_V_1.1 antibody specificity with two antibodies and** ***Scn1a*** **knockout (Na_V_1.1^−/−^) mice.** (A) Two examples showing triple staining with AnkG antibody, mouse anti-Na_V_1.1 (73-023, 1∶200, NeuroMab), and rabbit anti-Na_V_1.1 (AB5204, 1∶100, Millipore). Signals of the two Na_V_1.1 antibodies were both peaked at the proximal end of the AIS. Scale bar, 10 µm. (B–C) Double staining using antibodies for PV (green) and Na_V_1.1 (red) in tissue from wild-type (WT) mice (B) or homozygous Na_V_1.1 knockout (Na_V_1.1^−/−^) mice (C); lower panels show at larger magnification the neurons highlighted in the upper panels. Na_V_1.1 staining was evident at the AIS of PV neuron in WT mice (arrows), but was not detectable in any PV-containing neurites in the tissue obtained from Na_V_1.1^−/−^ mice. There were some little background signals, but they showed no correlation with neuronal structures. Scale bar, (upper panel) 20 µm and (lower panels) 10 µm.
(TIF)

Figure S7 **Verification of specificity of antibodies against Na_V_1.2 and Na_V_1.6 using antibodies of different origins and** ***Scn8a*** **knockout (Na_V_1.6^−/−^) mice.** (A–B) Triple staining of AnkG, Na_V_1.2, and Na_V_1.6 in cortical sections obtained from wild-type and Na_V_1.6^−/−^ mice. Note the absence of Na_V_1.6 staining in knockout mouse. (C) Double staining with AnkG and two different antibodies for Na_V_1.2 in rat cortical sections. Scale bar, 10 µm.
(TIF)

Figure S8 **AP threshold depends on the mixture level of Na^+^ channel subtype at the AIS.** (A) Simulation of activation (top) and inactivation (bottom) curves of Na^+^ currents generated by various mixtures of high and low Na^+^ channel subtypes. Red dots indicate half activation and inactivation potentials. (B) Half activation (top) and half inactivation (bottom) potentials became more positive as the percentage of high threshold Na^+^ channels increased in the simulated membrane patch. (C) Phase plots of APs in NEURON models with different ratios of high-low threshold Na^+^ channels at the AIS (top) and various AIS lengths (bottom). (D) AP threshold became more positive with increasing percentage of high-threshold channels at the AIS (top); however, AIS length variation made little difference in AP threshold.
(TIF)

Figure S9 **PaurTx3 (PTx3) selectively reduces the somatic Na^+^ current in SST and PC neurons.** (A) Puff application of 30 nM PTx3 (*n* = 6) showed no significant effect on Na^+^ currents obtained from somatic nucleated patches of PV neurons. (B) PTx3 significantly reduced somatic Na^+^ currents of SST neurons (*n* = 5). (C–E) Data from PCs. PTx3 significantly reduced Na^+^ currents evoked at somatic nucleated patches (C, *n* = 5) and outside-out patches excised from proximal AIS (D, *n* = 6) (presumably mediated by Na_V_1.2), but not Na_V_1.6-mediated currents obtained from isolated axon blebs of PCs (E, *n* = 5). Paired *t* test, * *p*<0.05; ** *p*<0.01. Error bars represent s.e.m.
(TIF)

## Abbreviations

α-DTx: α-Dendrotoxin
AIS: axon initial segment
AP: action potential
PC: pyramidal cell
PTx: picrotoxin
PTx3: phrixotoxin-3
PV: parvalbumin
SST: somatostatin
TTX: tetrodotoxin
