# Supplementary figures and images for: Action Potential Initiation in Neocortical Inhibitory Interneurons
Source: PLoS Biol. 2014 Sep 9;12(9):e1001944. doi: 10.1371/journal.pbio.1001944 (PMC4159120; doi:10.1371/journal.pbio.1001944)

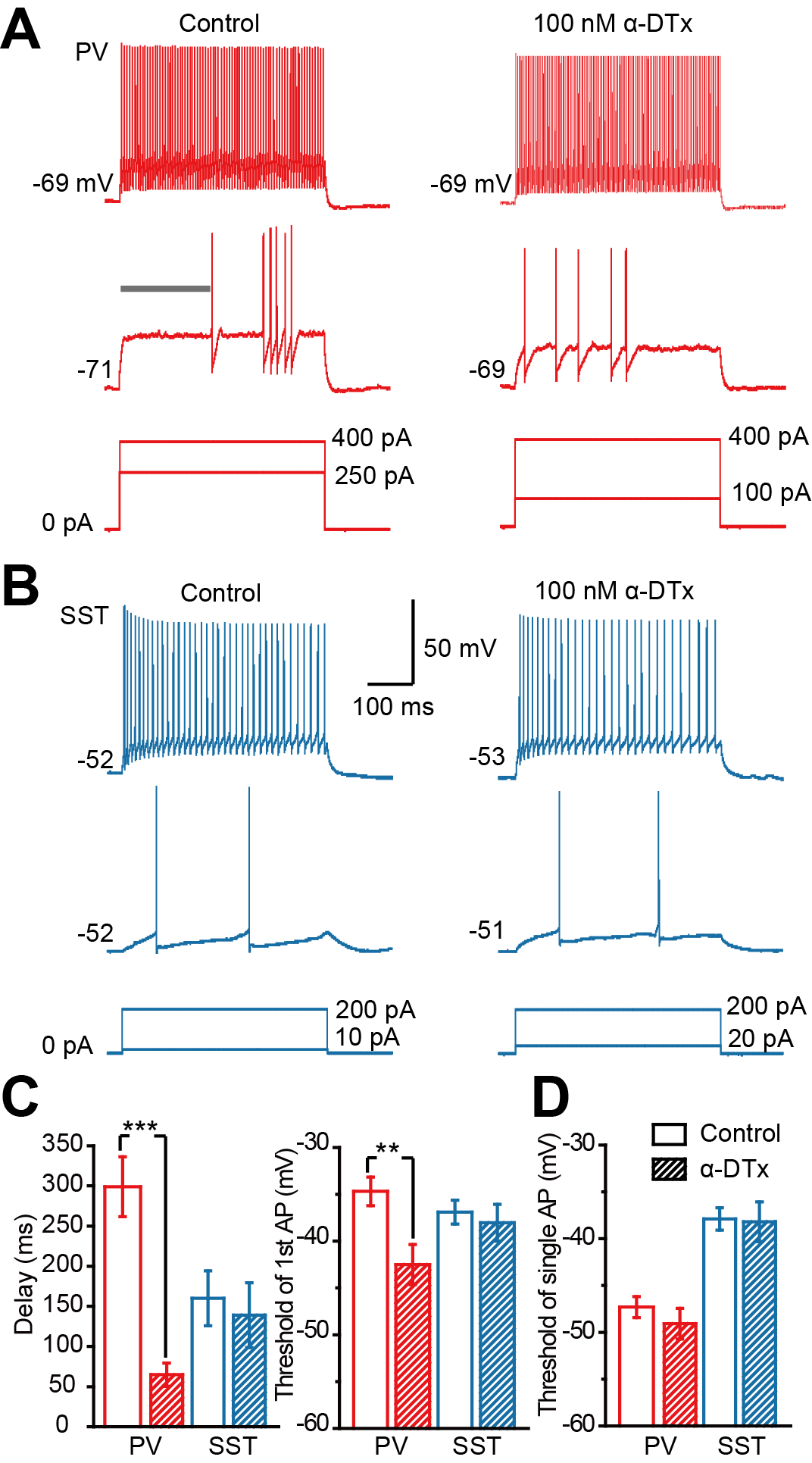

Supplement: Figure S1 — Blocking KV1 does not change the threshold of APs evoked by brief and strong stimulation. (A, Left) V m responses of a PV neuron to 250-pA (threshold current) and 400-pA step current injections. (Right) The same neuron after application of 100 nM α-DTx. The threshold current decreased to 100 pA, and the delay of the first AP (indicated by the gray bar) was diminished. (B, Left) V m responses of a SST neuron to 10-pA (threshold current) and 200-pA step current injections. (Right) The same neuron after application of 100 nM α-DTx. The duration of the depolarizating ramp before the first AP was not affected. (C, Left) Averaged delay of the first AP in both neuronal types before and after application of 100 nM α-DTx. Delay in SST neruons is the duration of the depolarizing ramp before the first AP. (Right) Voltage threshold changes of the first AP in both neuronal types before and after application of 100 nM α-DTx. (D) Voltage threshold changes of single APs induced by 2-ms current pulses in both neuronal types before and after α-DTx application. No significant difference was found between the control and α-DTx groups. *** p<0.001; ** p<0.01. Error bars represent s.e.m. (TIF) [file pbio.1001944.s001.tif]

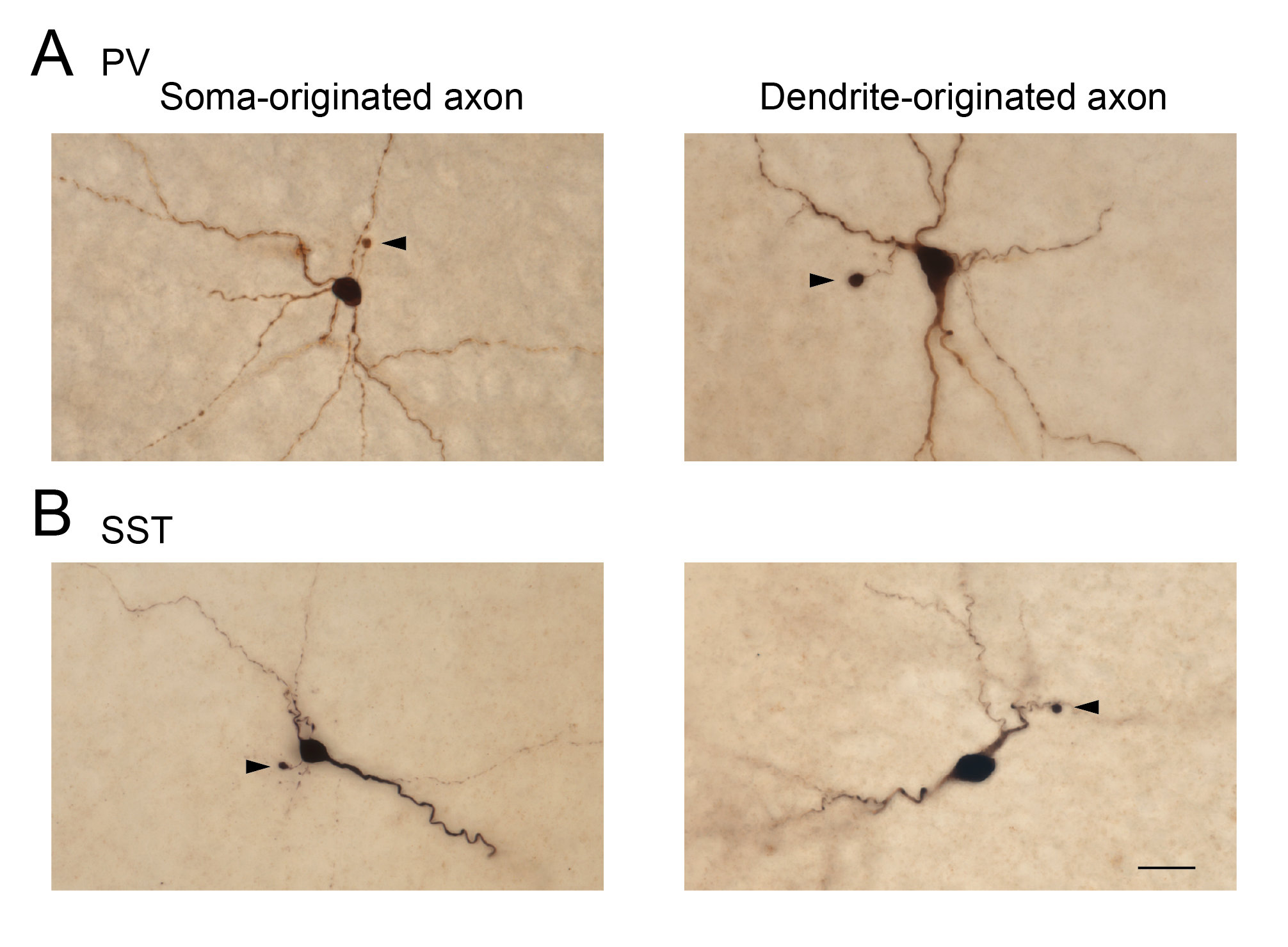

Supplement: Figure S2 — Soma- and dendrite-originated axons in PV and SST neurons. (A) PV neurons with axons originated from the soma (left) or the dendrite (right). (B) SST neurons. Arrowheads indicate the axonal blebs. The majority of PV and SST neurons emit their axons from the soma (86.7% of PV and 80.6% of SST neurons), whereas the remaining cells emit axons from dendrites. (TIF) [file pbio.1001944.s002.tif]

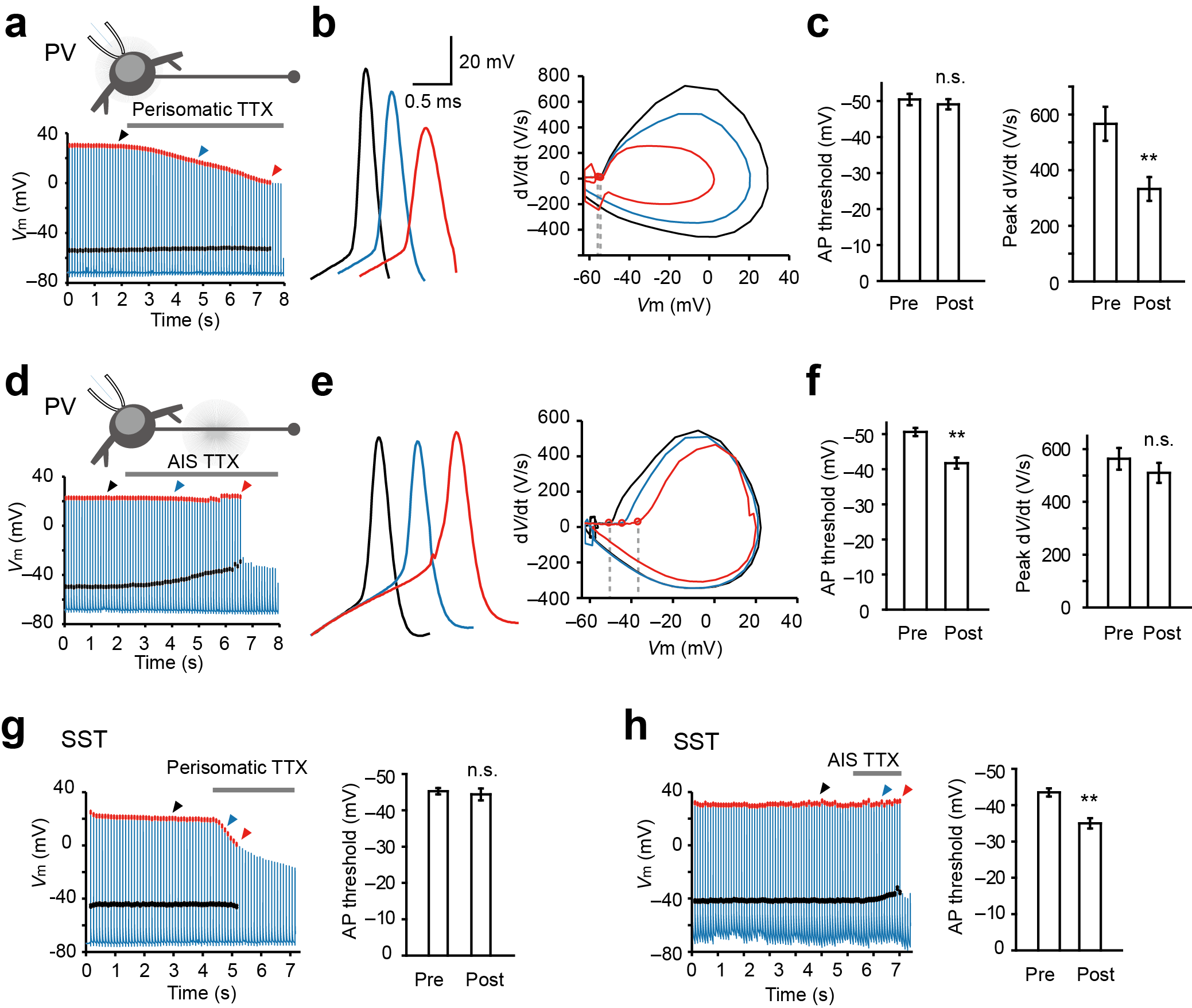

Supplement: Figure S3 — AIS Na+ channels determine the lowest AP threshold. (A) Example recording from a PV neuron showing the effect of perisomatic application of TTX (10 µM, puffing) on AP threshold and waveform. (Top) Schematic diagram showing the location of TTX application (gray area). (B) Perisomatic TTX showed no effect on AP thresholds but caused a dramatic decrease in peak amplitudes of APs. (Left) Three APs from (A) (arrowheads). (Right) Phase plot of APs. (C) Group data showing changes in AP threshold and peak amplitude of dV/dt. (D–F) Similar recording and analysis as in (A–C) except that TTX was applied at the AIS. TTX dramatically increased the AP thresholds but had no change in peak dV/dt. (G) In SST neurons, perisomatic TTX substantially decreased the peak amplitude of APs but not the threshold. (H) TTX application at the AIS of SST neurons significantly increased the AP thresholds. For statistical data, ** p<0.01. Error bars represent s.e.m. (TIF) [file pbio.1001944.s003.tif]

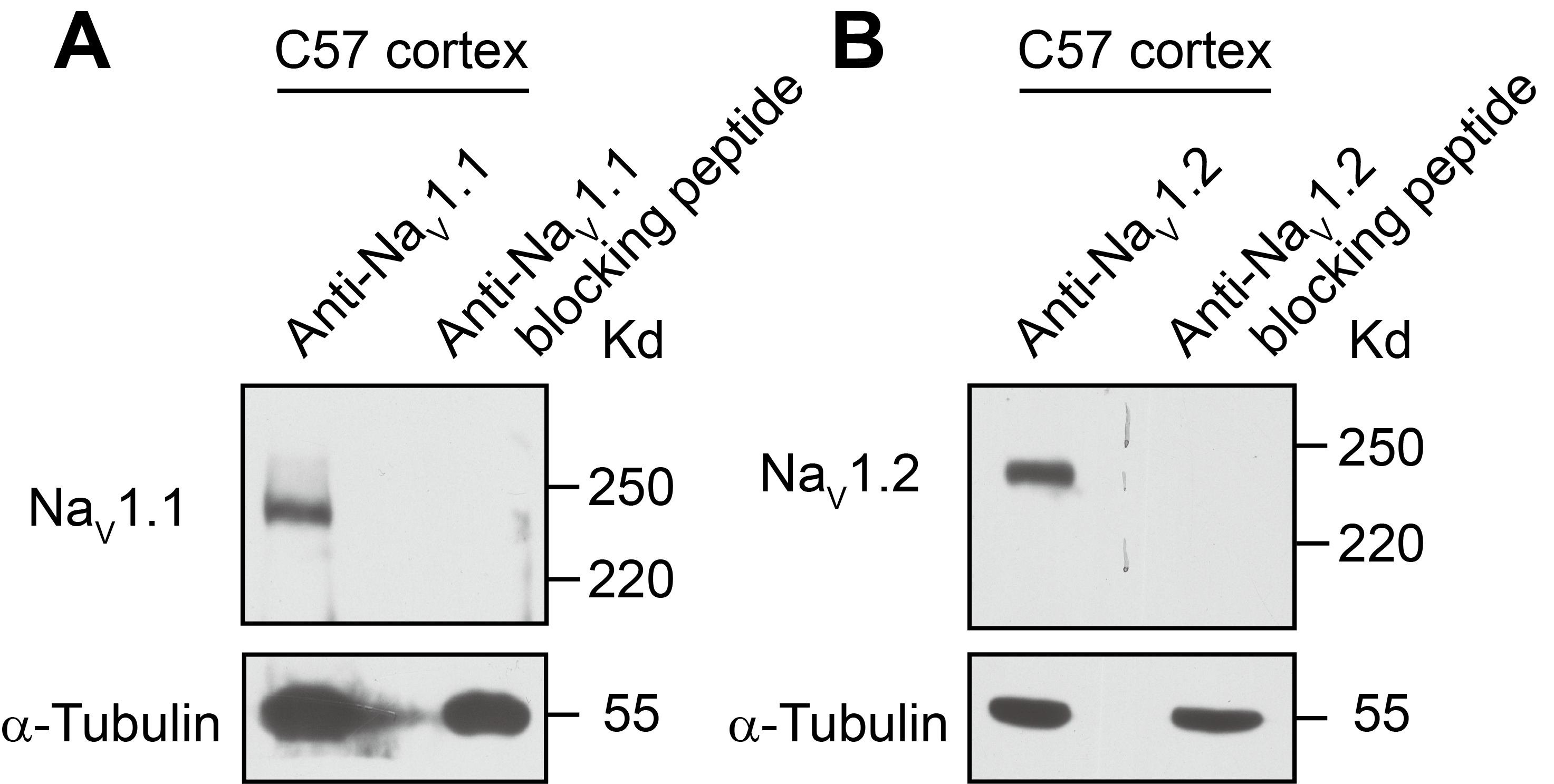

Supplement: Figure S4 — Verification of NaV1.1 and NaV1.2 antibodies with Western blot. Western blot analysis of the cortical extracts from C57 mice using (A) NaV1.1 and (B) NaV1.2 antibodies with or without pre-incubation of antigenic peptides. (TIF) [file pbio.1001944.s004.tif]

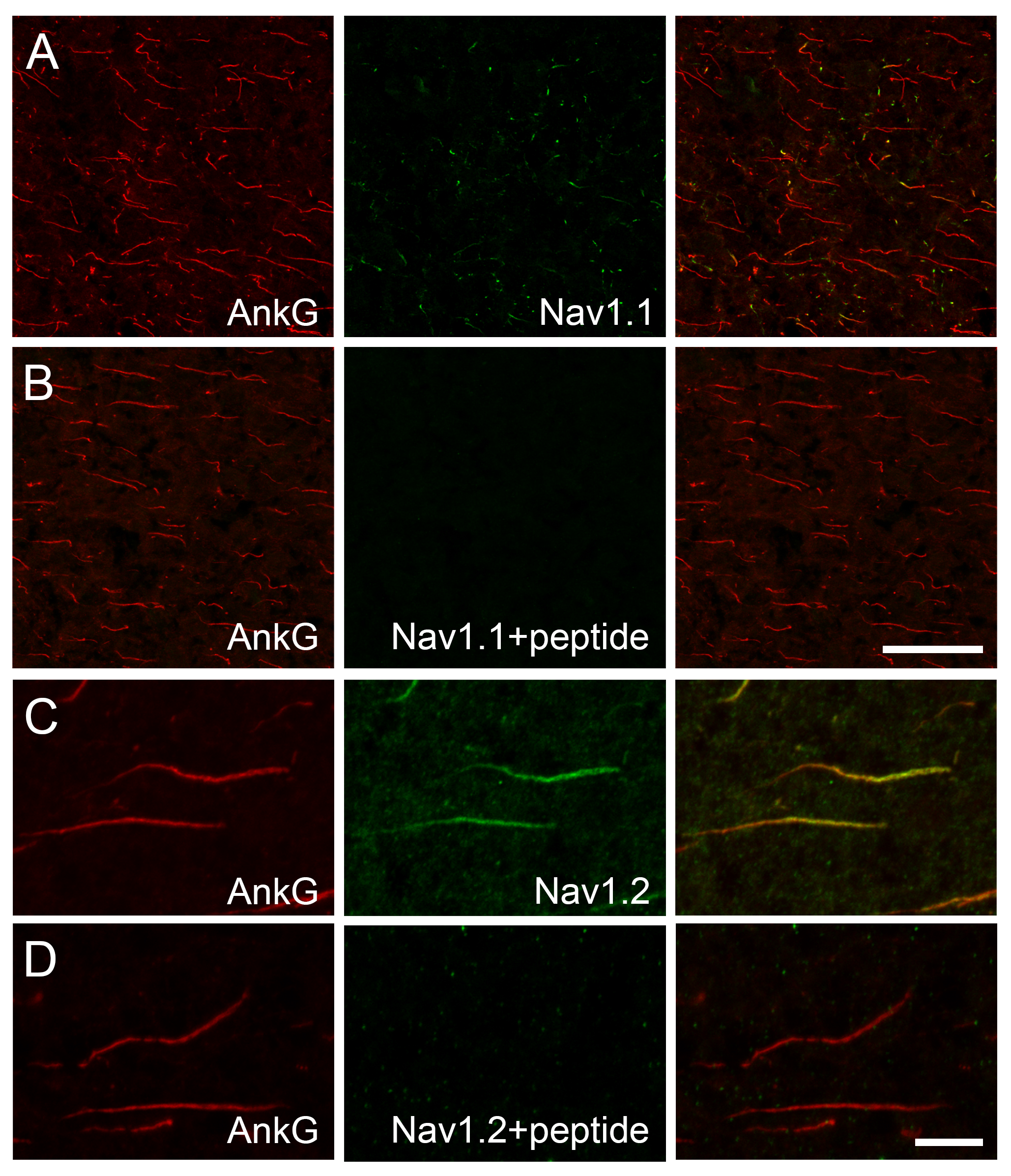

Supplement: Figure S5 — Immunosignal of NaV1.1 and NaV1.2 was eliminated by blocking peptides. (A) Double staining of AnkG and NaV1.1. (B) Double staining of AnkG and NaV1.1 in the presence of blocking peptide. (C) Double staining of AnkG and NaV1.2. (D) Double staining of AnkG and NaV1.2 in the presence of blocking peptide. Scale bar in (A–B), 50 µm; scale bar in (C–D), 10 µm. (TIF) [file pbio.1001944.s005.tif]

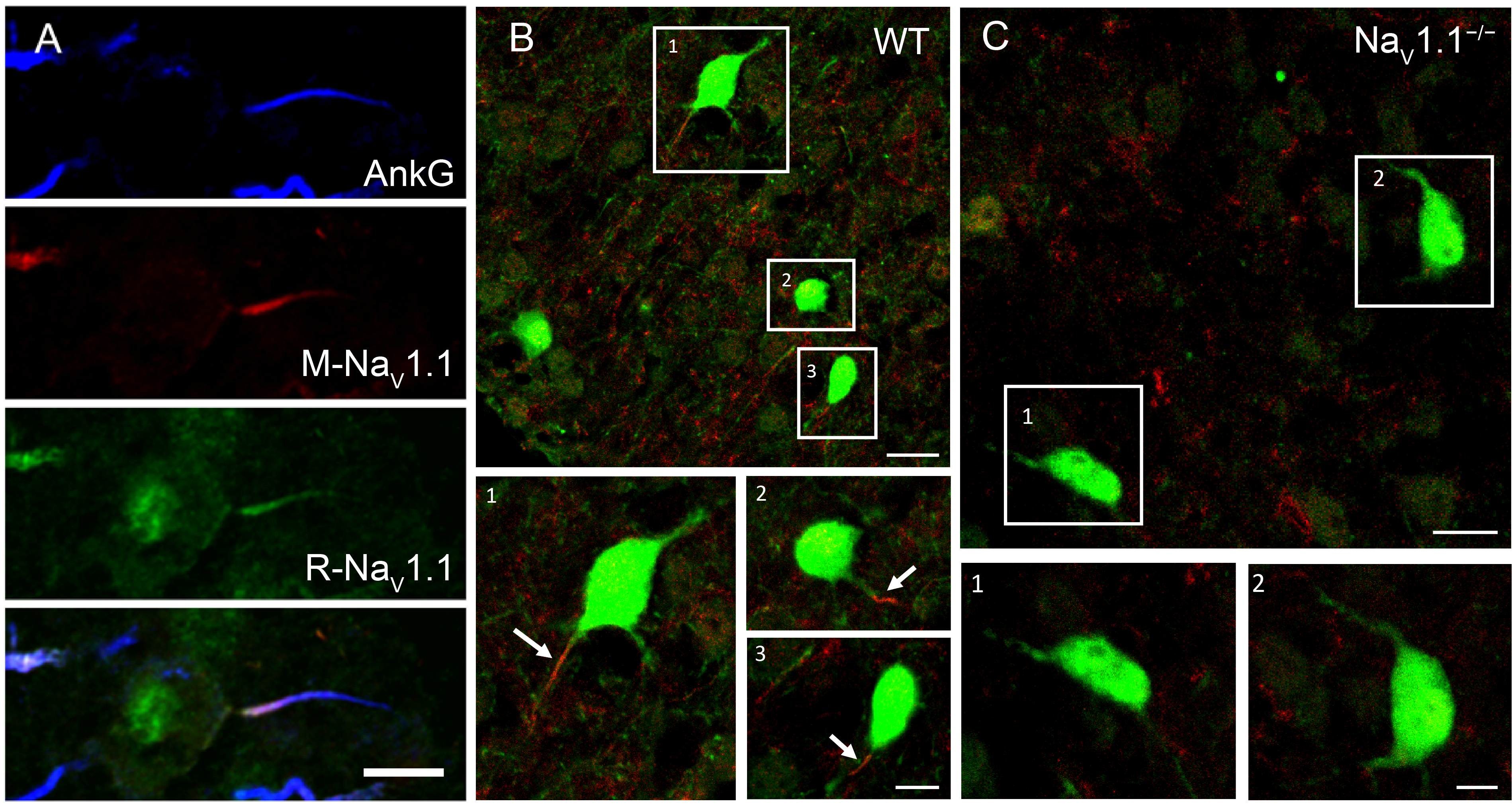

Supplement: Figure S6 — Verification of NaV1.1 antibody specificity with two antibodies and Scn1a knockout (NaV1.1−/−) mice. (A) Two examples showing triple staining with AnkG antibody, mouse anti-NaV1.1 (73-023, 1∶200, NeuroMab), and rabbit anti-NaV1.1 (AB5204, 1∶100, Millipore). Signals of the two NaV1.1 antibodies were both peaked at the proximal end of the AIS. Scale bar, 10 µm. (B–C) Double staining using antibodies for PV (green) and NaV1.1 (red) in tissue from wild-type (WT) mice (B) or homozygous NaV1.1 knockout (NaV1.1−/−) mice (C); lower panels show at larger magnification the neurons highlighted in the upper panels. NaV1.1 staining was evident at the AIS of PV neuron in WT mice (arrows), but was not detectable in any PV-containing neurites in the tissue obtained from NaV1.1−/− mice. There were some little background signals, but they showed no correlation with neuronal structures. Scale bar, (upper panel) 20 µm and (lower panels) 10 µm. (TIF) [file pbio.1001944.s006.tif]

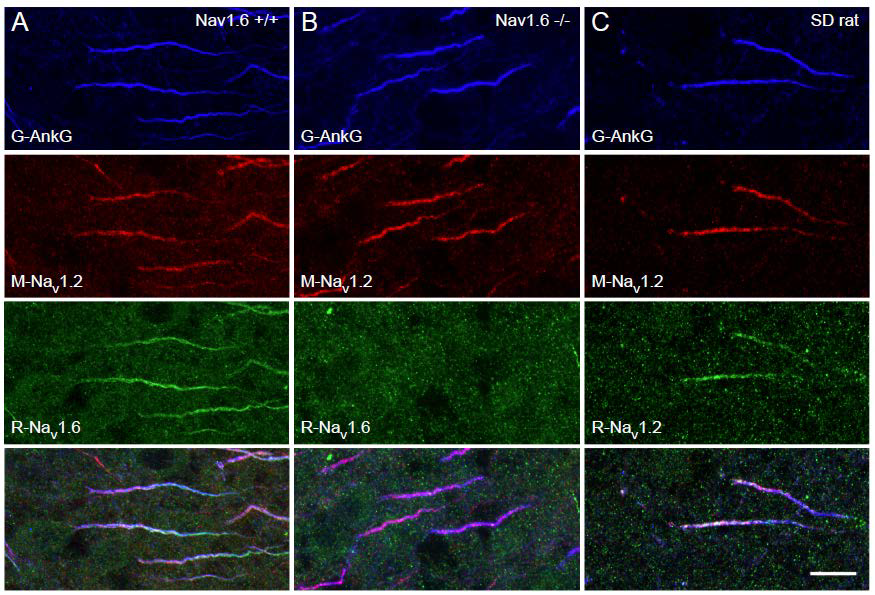

Supplement: Figure S7 — Verification of specificity of antibodies against NaV1.2 and NaV1.6 using antibodies of different origins and Scn8a knockout (NaV1.6−/−) mice. (A–B) Triple staining of AnkG, NaV1.2, and NaV1.6 in cortical sections obtained from wild-type and NaV1.6−/− mice. Note the absence of NaV1.6 staining in knockout mouse. (C) Double staining with AnkG and two different antibodies for NaV1.2 in rat cortical sections. Scale bar, 10 µm. (TIF) [file pbio.1001944.s007.tif]

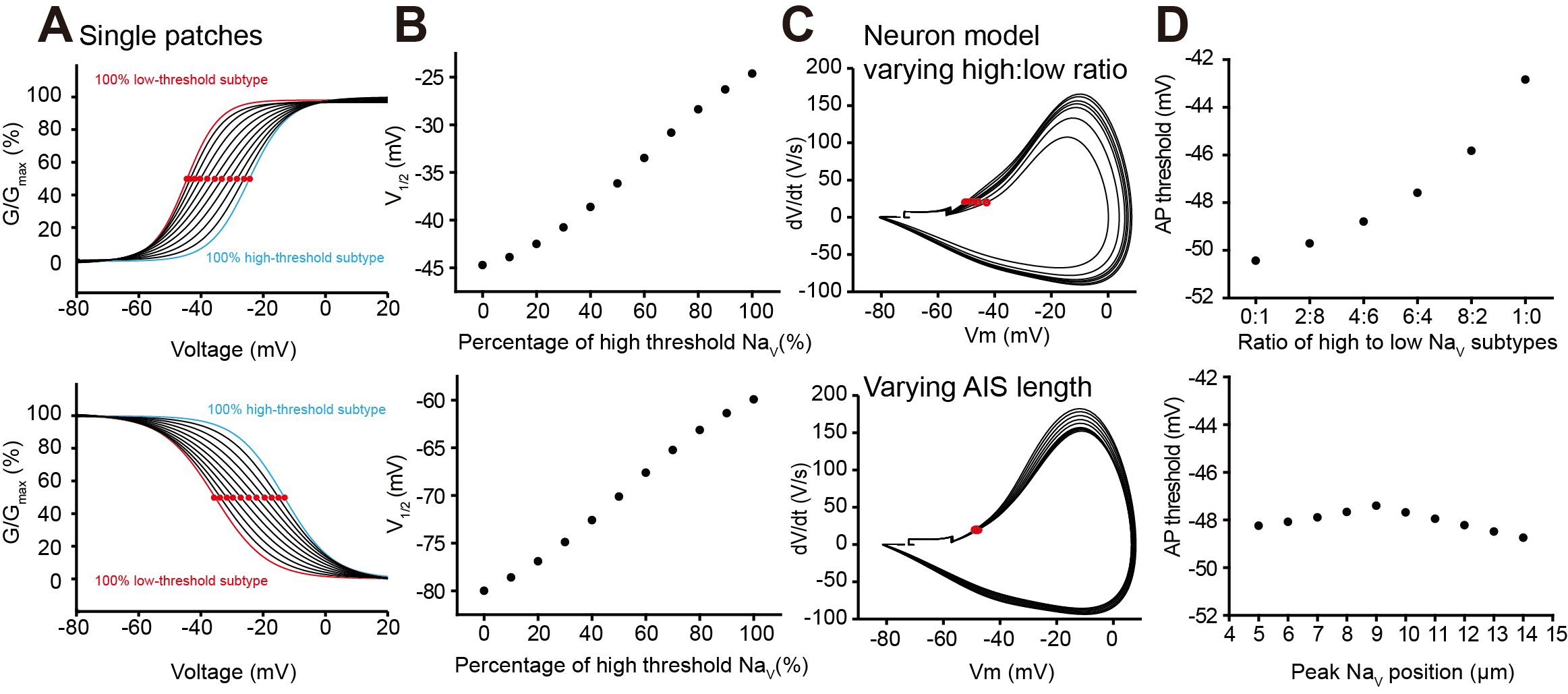

Supplement: Figure S8 — AP threshold depends on the mixture level of Na+ channel subtype at the AIS. (A) Simulation of activation (top) and inactivation (bottom) curves of Na+ currents generated by various mixtures of high and low Na+ channel subtypes. Red dots indicate half activation and inactivation potentials. (B) Half activation (top) and half inactivation (bottom) potentials became more positive as the percentage of high threshold Na+ channels increased in the simulated membrane patch. (C) Phase plots of APs in NEURON models with different ratios of high-low threshold Na+ channels at the AIS (top) and various AIS lengths (bottom). (D) AP threshold became more positive with increasing percentage of high-threshold channels at the AIS (top); however, AIS length variation made little difference in AP threshold. (TIF) [file pbio.1001944.s008.tif]

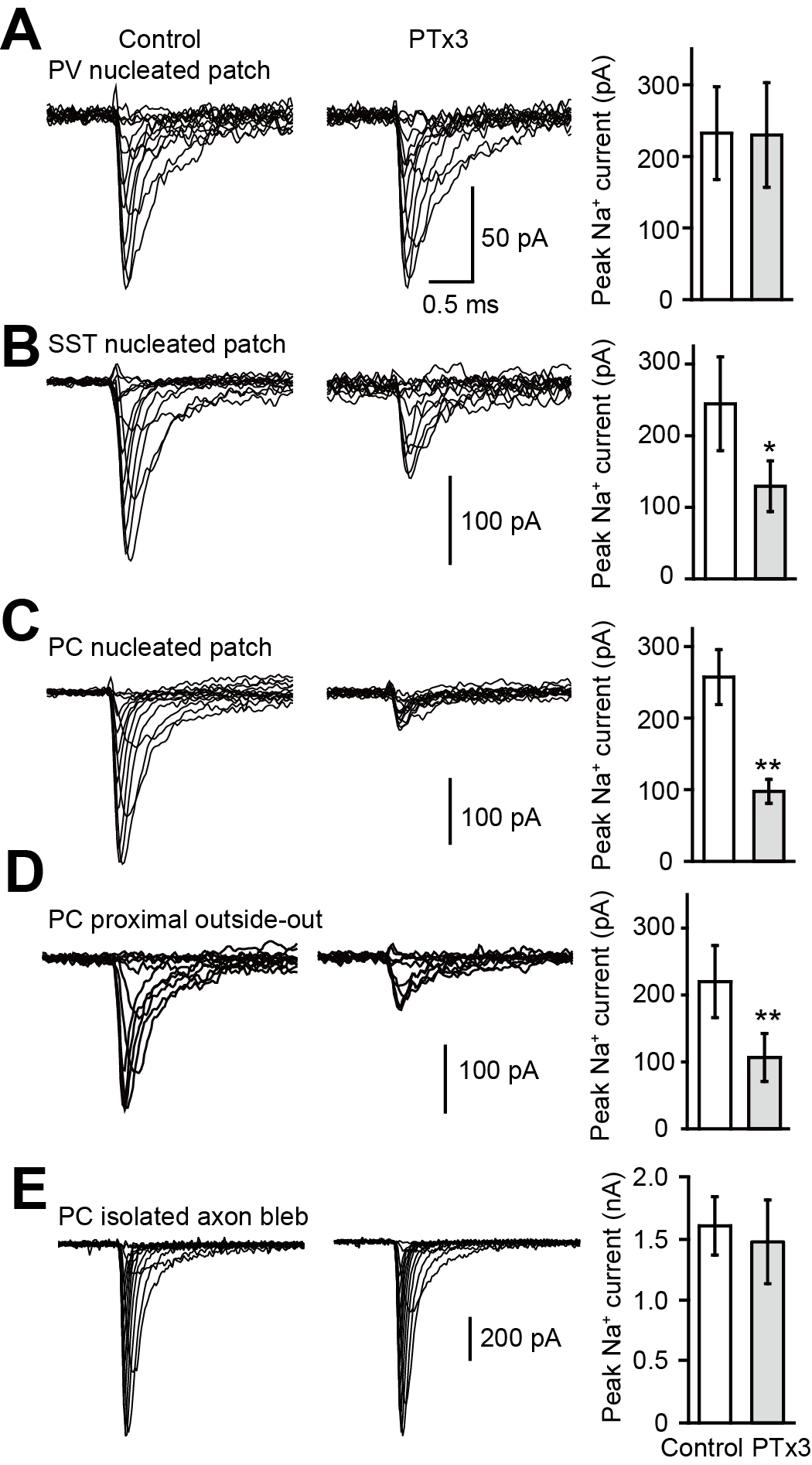

Supplement: Figure S9 — PaurTx3 (PTx3) selectively reduces the somatic Na+ current in SST and PC neurons. (A) Puff application of 30 nM PTx3 (n = 6) showed no significant effect on Na+ currents obtained from somatic nucleated patches of PV neurons. (B) PTx3 significantly reduced somatic Na+ currents of SST neurons (n = 5). (C–E) Data from PCs. PTx3 significantly reduced Na+ currents evoked at somatic nucleated patches (C, n = 5) and outside-out patches excised from proximal AIS (D, n = 6) (presumably mediated by NaV1.2), but not NaV1.6-mediated currents obtained from isolated axon blebs of PCs (E, n = 5). Paired t test, * p<0.05; ** p<0.01. Error bars represent s.e.m. (TIF) [file pbio.1001944.s009.tif]
